# Evaluation of the SPARTACUS-Urban Radiation Model for Vertically Resolved Shortwave Radiation in Urban Areas

**DOI:** 10.1007/s10546-022-00706-9

**Published:** 2022-06-29

**Authors:** Megan A. Stretton, William Morrison, Robin J. Hogan, Sue Grimmond

**Affiliations:** 1 Department of Meteorology, University of Reading, Reading, UK; 2 European Centre for Medium-Range Weather Forecasts, Reading, UK

**Keywords:** The DART model, Shortwave radiation, The SPARTACUS-Urban model, Urban form

## Abstract

The heterogenous structure of urban environments impacts interactions with radiation, and the intensity of urban–atmosphere exchanges. Numerical weather prediction (NWP) often characterizes the urban structure with an infinite street canyon, which does not capture the three-dimensional urban morphology realistically. Here, the SPARTACUS (Speedy Algorithm for Radiative Transfer through Cloud Sides) approach to urban radiation (SPARTACUS-Urban), a multi-layer radiative transfer model designed to capture three-dimensional urban geometry for NWP, is evaluated with respect to the explicit Discrete Anisotropic Radiative Transfer (DART) model. Vertical profiles of shortwave fluxes and absorptions are evaluated across domains spanning regular arrays of cubes, to real cities (London and Indianapolis). The SPARTACUS-Urban model agrees well with the DART model (normalized bias and mean absolute errors < 5.5%) when its building distribution assumptions are fulfilled (i.e., buildings randomly distributed in the horizontal). For realistic geometry, including real-world building distributions and pitched roofs, SPARTACUS-Urban underestimates the effective albedo (< 6%) and ground absorption (< 16%), and overestimates wall-plus-roof absorption (< 15%), with errors increasing with solar zenith angle. Replacing the single-exponential fit of the distribution of building separations with a two-exponential function improves flux predictions for real-world geometry by up to half. Overall, SPARTACUS-Urban predicts shortwave fluxes accurately for a range of geometries (cf. DART). Comparison with the commonly used single-layer infinite street canyon approach finds SPARTACUS-Urban has an improved performance for randomly distributed and real-world geometries. This suggests using SPARTACUS-Urban would benefit weather and climate models with multi-layer urban energy balance models, as it allows more realistic urban form and vertically resolved absorption rates, without large increases in computational cost or data inputs.

**Supplementary Information:**

The online version contains supplementary material available at 10.1007/s10546-022-00706-9.

## Introduction

Given their high concentrations of both people and infrastructure, cities are places of high vulnerability to variations in weather, climate, and air quality (Baklanov et al. 2018). Currently, limited-area numerical-weather-prediction (NWP) models have spatial resolutions such that cities span multiple model grid boxes (e.g., Hagelin et al. (2017)). In addition, global NWP models generally have poor representation of urban structure and energy exchanges, for example the European Centre for Medium-Range Weather Forecasts IFS (Integrated Forecasting System) model with 9–18 km resolution, with no urban model (Hogan et al. 2017). As NWP spatial resolution increases, smaller-scale processes will need to be resolved. Alongside this, global population and urban land cover are expected to increase, bringing about a need for greater understanding of energy exchanges between the surface and the atmosphere (Loridan and Grimmond 2012).

Cities have complex three-dimensional structures, with varying building heights, densities, materials, arrangements, shapes, and surroundings. These affect the radiative exchanges and heat storage within the urban surface (Grimmond et al. 2010; Yang and Li 2015; Ao et al. 2016), e.g., altering the effective shortwave albedo due to multiple reflections in the street canyon (Aida and Gotoh 1982). Given that shortwave radiation is the most important contribution to the surface energy balance, it is vital to understand how it is absorbed within urban areas (Fortuniak 2008).

Models have been developed to account for the three-dimensional nature of urban surfaces. A common approach is to simplify the urban form as a canyon of infinite length between buildings that are of equal height with a fixed height-to-width (*H/W*) ratio (Nunez and Oke 1977). This urban-canyon approach is fast enough for NWP, and has been applied to urban radiation specifically (e.g., Aida 1982; Arnfield 1982a, 1988; Harman et al. 2004) and other energy balance fluxes (e.g., Masson 2000; Kusaka et al. 2001a; Lee and Park 2008). This approach subdivides the canyon into three facets: walls, roof, and ground. Advancements of this approach include models subdividing facets further into sunlit and shaded, with varying canyon orientation (e.g., Oleson et al. 2008a, b), accounting for different building heights (Martilli et al. 2002), and the interactions between neighbouring canyons (Schubert et al. 2012). Some models have added vegetation, both at ground level and in the vertical plane (e.g., street trees) (Lemonsu et al. 2012; Krayenhoff et al. 2014; Redon et al. 2017).

These improvements in the vertical structure of urban form have led to improvements in the prediction of shortwave fluxes onto roofs (Schubert et al. 2012). Despite this, many models still make the unrealistic assumption of an infinitely long urban canyon. This leads to models neglecting key features of the urban form, such as intersections, building height variations, courtyards, and clusters of buildings. Ignoring these features impacts building shadowing, radiation trapping between buildings, and increased penetration of shortwave radiation to the surface in open areas such as parking areas, hence impacting the overall energy balance.

Building-resolving models, with details of each individual building and facet, are suitable for microscale research applications but not NWP, given their high data and computational demands (e.g., Krayenhoff and Voogt 2007; Krayenhoff et al. 2015; Resler et al. 2017). These models simulate radiative interactions between individual buildings, requiring detailed three-dimensional (3D) geometry and material data, which are hard to obtain for large areas (Ghandehari et al. 2018; Masson et al. 2020). Gastellu-Etchegorry et al. (2012) suggested a key application for complex building-resolving models is both to calibrate and evaluate simpler radiative transfer models (e.g., suitable for NWP). However, very few urban radiative transfer models have been evaluated against these models. One exception is the evaluation of shortwave radiation in the Town Energy Balance (TEB) model against the SOLENE explicit radiative transfer model, considering vertical vegetation (Redon et al. 2017), although an infinite street canyon is used in both the TEB and SOLENE models. Other urban radiative transfer models suitable for NWP could (or should) be calibrated and/or evaluated using explicit 3D models.

Here, we evaluate the shortwave performance of the SPARTACUS (Speedy Algorithm for Radiative Transfer through Cloud Sides) radiative transfer model for urban areas, SPARTACUS-Urban (Hogan 2019a), with respect to the more detailed explicit 3D Discrete Anisotropic Radiative Transfer (DART) model (Gastellu-Etchegorry 2008). The SPARTACUS-Urban model resolves the vertical structure of the urban canopy and exploits the recent finding that wall-to-wall separation distances in an urban area fit an exponential distribution well (Hogan 2019b). It can account for atmospheric absorption, emission, and scattering in the urban environment, rather than assuming a vacuum, as is done by most urban radiation models (e.g., Masson 2000; Harman et al. 2004), while being fast enough for use in NWP. Although it can represent vertical profiles of vegetation, this capability is not evaluated here. We examine SPARTACUS-Urban’s ability to predict the vertical profile of the clear-air downwelling and upwelling fluxes, and the absorption into walls and roofs, across a range of urban forms: from simple cuboid ‘buildings’ to highly realistic structures.

The methods include a description of the SPARTACUS-Urban (Sect. 2) and DART (Sect. 3) models, and of the evaluation techniques (Sect. 4). After an investigation of the underpinning assumptions that SPARTACUS-Urban makes about the urban form (Sect. 5), the results of the evaluation are presented (Sect. 6). Finally, a comparison of SPARTACUS-Urban with DART is made with respect to the Harman et al. (2004) method for radiation within an urban street canyon (Sect. 7).

## Description of the SPARTACUS-Urban Model

The SPARTACUS-Urban model (Hogan 2019a) uses an approach that originates from the SPARTACUS model for simulating 3D radiative transfer in complex cloud fields (Hogan and Shonk 2013; Hogan et al. 2016) and SPARTACUS-Vegetation for 3D interaction of radiation in forest-type vegetation (Hogan et al. 2018). These algorithms share a common mathematical approach for treating radiative transfer in the presence of objects that are randomly distributed in the horizontal. The SPARTACUS-Surface open-source software package (Hogan 2021) combines the capabilities of SPARTACUS-Urban and SPARTACUS-Vegetation, but since vegetation is not considered here we refer to the algorithm as SPARTACUS-Urban.

The SPARTACUS-Urban model is underpinned by the one-dimensional discrete ordinate method (Stamnes et al. 1988), which assumes that diffuse radiation travels in 2*N* streams of different elevations, with *N* streams per hemisphere. As *N* increases, the radiation field is described more accurately, but with increased computational cost. Here 16 streams (*N* = 8) are used. The SPARTACUS-Urban model splits a scene (defined here as any combination of building geometry, solar zenith angle, and albedo) vertically by height, *z*, into *n* horizontal layers above an assumed flat ground level. Each layer is split horizontally into ‘regions’ of clear sky, vegetation, or buildings. As with other urban models, SPARTACUS-Urban computes the interaction of radiation with three urban facets (wall, roof, ground) and optionally vegetation.

Through the process of this work, we have found that two modifications to SPARTACUS-Urban are needed. Section 2.1 describes how to treat the more common occurrence of large open spaces such as parking areas and parks than predicted by the exponential model of urban geometry used by SPARTACUS-Urban. Section 2.2 describes a correction to account for fine structure in the building perimeters.

### Modification to Treat Non-Exponential Building Separations

To characterize urban geometry, SPARTACUS-Urban takes building plan area fraction (*λ*_*p*_) and the normalized building perimeter length (*L*) as a function of *z* for the city area of interest (hereafter “domain”). Thus, *L*(*z*) is the total building perimeter normalized by the horizontal area of the domain (units m^−1^). The SPARTACUS-Urban model discretizes the vertical profile into *n* layers where layer *i* has thickness Δ*z*_*i*_ and normalized perimeter length *L*_*i*_. Following this, the normalized wall area (*A*_*W*_) is

1 $$ \begin{array}{*{20}c} {A_{W} = \mathop \sum \limits_{i}^{n} L_{i} \Delta z_{i} = \pi \lambda_{f} ,} \\ \end{array} $$

which is proportional to the frontal area index (*λ*_*f*_) or projected wall area for a particular azimuthal direction (Raupach and Shaw 1982; Grimmond and Oke 1999; Sützl et al. 2020). The SPARTACUS-Urban model assumes that walls face in all azimuthal directions with equal probability.

Based on analysis of the geometry in real cities (Hogan 2019b), SPARTACUS-Urban assumes that the probability distribution of wall-to-wall horizontal separation distances, *p*_*ww*_(*x*), and the distribution of ground-to-wall separations, *p*_*gw*_(*x*), each follow an exponential distribution:

2 $$ \begin{array}{*{20}c} {p_{{{\text{ww}}}} \left( x \right) = p_{{{\text{gw}}}} \left( x \right) = \frac{1}{X}{\text{exp}}\left( { -\frac{x}{X}} \right),} \\ \end{array} $$

where *x* is the horizontal wall-to-wall distance in any azimuthal direction, and *X* is the mean wall-to-wall distance, or ‘e-folding’ distance (Hogan 2019a, b). This exponential distribution allows the direct and diffuse streams of radiation to be attenuated according to the Beer–Lambert law (Hogan 2019a).

The assumption that buildings are randomly distributed horizontally has two other important consequences. First, the horizontal distribution of radiation between buildings (or vegetation, if included) need not be explicitly simulated; rather the horizontal-mean radiation field in each direction is computed as a function of height alone. Second, at a given height, the rate of interception of radiation by buildings is proportional the total building perimeter. However, in real cities Eq. 2 tends to underpredict the frequency of large building separations such as parks, parking-areas, and plazas (e.g., Fig. 6, Hogan (2019b)). Hence, the penetration of direct shortwave radiation to street level tends to be underpredicted when the sun is low in the sky, leading to an overprediction in absorption of shortwave radiation by walls. To address this, we replace Eq. 2 with the sum of two exponentials, with a weighting between them (*G*_ww_):

3 $$ \begin{array}{*{20}c} {p_{{{\text{ww}}}} \left( x \right) = \frac{{G_{{{\text{ww}}}} }}{{X_{1} }}\exp \left( { -\frac{x}{{X_{1} }}} \right) + \frac{{1 -G_{{{\text{ww}}}} }}{{X_{2} }}\exp \left( { -\frac{x}{{X_{2} }}} \right).} \\ \end{array} $$

where *X*_*1*_ and *X*_*2*_ are the e-folding distance of each exponential. The weighting function (Eq. 3) better predicts the frequency of large building separations by up to three times that of Eq. 2, in theory improving the prediction of radiative fluxes. Applying Eq. 1 of Hogan (2019b) leads to an equation for *p*_*gw*_(*x*) with both the same form as Eq. 3 and the same *X*_1_ and *X*_2_ values, but a different weighting coefficient *G*_*gw*_

4 $$ \begin{array}{*{20}c} {G_{{{\text{gw}}}} = \frac{{G_{{{\text{ww}}}} X_{1} }}{{G_{{{\text{ww}}}} X_{1} + \left( {1 -G_{{{\text{ww}}}} } \right)X_{2} }} .} \\ \end{array} $$

To use the two-exponential model within SPARTACUS-Urban, we modify *L*(*z*) so that it varies with *θ*_*0*_, where *θ*_*k*_ is the zenith angle of a stream *k*, where *k* = 0 indicates the solar zenith angle, defining an effective normalized perimeter length, $\hat{L}$, given by

5 $$ \begin{array}{*{20}c} {\hat{L} = \frac{{\pi \left( {1 -\lambda_{p} } \right)}}{{\hat{X}}} .} \\ \end{array} $$

This is identical to Eq. 8 of Hogan (2019b) except for the use of an effective e-folding building separation ($\hat{X})$ in place of the e-folding building separation used to characterize the horizontal distribution of urban geometry. To derive an analytical relation between $\hat{L}$ and the two-exponential fit coefficients (*G*_gw_, *X*_1_ and *X*_2_), we assume that if all buildings had the same height, *H*, the fraction of direct solar radiation penetrating to street level (*F*_0*g*_) can be predicted exactly using Eq. 3 of Hogan (2019b)

6 $$ {F_{0g} = \mathop \int \limits_{{x_{0} }}^{\infty } p_{{{\text{gw}}}} \left( x \right){\text{d}}x,}  $$

where *x*_0_ = *H* tan *θ*_*0*_. As buildings are typically not all the same height, we use the mean building height ($\overline{H})$. Substituting our Eq. 3 (but for *p*_*gw*_) in Eq. 6 gives

7 $$ \begin{array}{*{20}c} {F_{0g} = G_{{{\text{gw}}}} \exp \left( { -\frac{{x_{0} }}{{X_{1} }}} \right) + \left( {1 -G_{{{\text{gw}}}} } \right){\text{ exp}}\left( { -\frac{{x_{0} }}{{X_{2} }}} \right).} \\ \end{array} $$

However, as SPARTACUS-Urban follows an exponential building distribution (Eq. 2) we apply Eq. 6, leading to

8 $$ \begin{array}{*{20}c} {F_{0g} = \exp \left( { -\frac{{x_{0} }}{{\hat{X}}}} \right) .} \\ \end{array} $$

This is equal to Eq. 20 of Hogan (2019b), but using the effective e-folding building separation, $\hat{X}$. Combining this with Eq. 5 gives

9 $$ \begin{array}{*{20}c} {\hat{L}_{0} = -\frac{{\pi \left( {1 -\lambda_{p,0} } \right)\ln \left( {F_{0g} } \right)}}{{x_{0} }}, } \\ \end{array} $$

where $\hat{L}$_0_ is $\hat{L}$ at the surface. This can be applied to any city with building footprint data, but probability distributions can only be computed using the Hogan (2019b) method near the surface for building densities, *λ*_*p*_ > 0.01. Using $\hat{L}$_0_ we scale *L*(*z*) at each height using the building cover fraction at that height

10 $$ \begin{array}{*{20}c} {\hat{L}\left( z \right) = L\left( z \right)\left( {\frac{{\hat{L}_{0} \lambda_{p} \left( z \right)}}{{L_{0} \lambda_{p,0} }} + \frac{{\lambda_{p,0} -\lambda_{p} \left( z \right)}}{{\lambda_{p,0} }}} \right).} \\ \end{array} $$

This leads to the scaling factor to *L*(*z*) (Eq. 10) having a greater impact near the surface where *λ*_*p*_ is larger, and a reduced impact as buildings thin out towards the top of any urban canopy, where $\hat{L}$ tends toward *L*. The appropriateness of the single- and two-exponential methods in describing urban environments is discussed in Sect. 5, and their use in calculating fluxes is assessed in Sect. 6.3.

### Modification to Treat Building Concavity

From SPARTACUS-Urban’s use of *L* to describe the rate of interception of radiation by building walls, it follows that the width of the shadow cast by any building (*W*_*S*_), averaged over all azimuthal illumination directions, is assumed to be equal to $P/\pi$ where *P* is the building’s perimeter length. This is true for convex shapes such as cylinders and cuboids, but not for many real-world buildings, which have fine structure in their perimeters, and so the shadows cast in SPARTACUS-Urban tend to be wider than reality. We therefore define the “concavity parameter” *C* as the ratio of the true perimeter length to the perimeter length of the equivalent convex hull, which for an individual building is given by

11 $$ \begin{array}{*{20}c} {C = \frac{P}{{\pi W_{S} }} .} \\ \end{array} $$

Values of *C* are found to be 1 or greater, and to vary with height (Appendix 1, Fig. 12 and Online Resource 7). The effective concavity parameter of all the buildings at a particular height can be calculated by replacing the numerator and denominator of Eq. 11 each by the average over all buildings. In this work, the median of *C* at all heights above $\overline{H}$ for each domain is used as a scaling factor to *L*(*z*) at all heights (i.e., $L\left( z \right)/C$).

## Description of the Discrete Anisotropic Radiative Transfer Model

The three-dimensional DART model (Gastellu-Etchegorry et al. 2015; Landier et al. 2018) simulates radiation propagation for heterogenous scenes that can include vegetation, buildings, a within-canopy atmosphere, and variations in ground height (i.e., topography). The latter can be imported using 3D vector models. The DART model has been evaluated using observations and other 3D radiative transfer models for vegetation (Sobrino et al. 2011; Widlowski et al. 2015), and has been applied in urban areas (e.g., Landier et al. 2018; Chrysoulakis et al. 2018; Morrison et al. 2020a).

Radiative fluxes are calculated iteratively, with radiation tracked and emitted along a number of discrete directions within angular cones (Gastellu-Etchegorry et al. 2015) using a 3D array of voxels to facilitate radiation tracking. Radiation interacts with the scene elements in each voxel. Per-voxel scattered, absorbed, emitted, upwelling and downwelling radiative fluxes are updated after each iteration, with upwelling and downwelling fluxes for each voxel stored in the top face of each voxel. Here, we use DART’s ability to calculate the radiative budget to assess the SPARTACUS-Urban emission and absorption of shortwave radiation.

## Evaluation Methods

### Model Domains

**Fig. 1:**
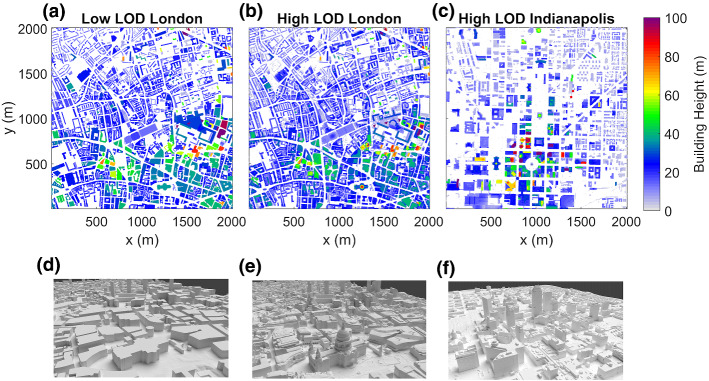
Real-world domains in parts of (**a**, **b**, **d**–**e**) London, and (**c**, **f**) Indianapolis; for the following LOD: (**a**, **d**) low, and (**b**, **c**, **e**, **f**) high; with building height for (**a**–**c**) SPARTACUS-Urban—rasterized building footprints, (**d**–**f**) DART- 3D building model. Data sources are given in Table 1

Four types of urban form (F) (Table 1) are used in the evaluation, from simplest to most complex:

1. Regular array of cubes that repeat on a regular grid (F_REG1_ and F_REG2_, Table 1). Cubes are often used to approximate urban processes (e.g., Aida 1982; Kondo et al. 2005; Kanda et al. 2005; Kanda 2007; Morrison et al. 2018) as they create a regular grid street pattern that occurs in many cities in the U.S. and China (Figueiredo and Amorim 2007; Han et al. 2020).
2. Random cuboids (F_RAND_) where building centroids are randomly located within a domain with random building heights, widths, and orientations. Twelve domains are used, but four are focussed on, with building fraction at the surface (*λ*_*p,0*_) and $\overline{H }$ values spanning those found in areas of real cities (F_RAND1_–F_RAND4_, Table 1) based on prior studies (Loridan and Grimmond 2012). This form type tests situations where the SPARTACUS-Urban building layout assumptions are met. These might be more typical of European cities, where street orientation is more random.
3. Low level-of-detail (LOD) real-world geometry using building footprint data, with one height per building creating flat roofs and walls that do not taper, and flat ground (Fig. 1a, d). Building footprints used are for part of central London (F_Lon,L_, Table 1).
4. High LOD real-world geometry where heights can vary across a building (Fig. 1b, e). Parts of two cities are analyzed: a dense European megacity London (F_Lon,H_)—and an open low-density U.S. grid-city—Indianapolis (F_Ind,H_).

**Table 1:** Different urban forms (F, code subscripts) that are simulated with different: horizontal raster resolution (Δ*x*), vertical height intervals (Δ*z*), domain sizes, building fraction at the ground (*λ*_*p,0*_), normalized building perimeter length at the ground (*L*_*0*_), and mean building height ($\overline{H}$)

Type	Form (F) code	Δ*x* (m)	Δ*z* (m)	Domain size (m^2^)	*λ*_*p,0*_	*L*_*0*_ (m^−1^)	$\overline{H}$(m)	Derived from
Regular cubes	F_REG1_	0.5	0.5	20 × 20	0.0625	0.050	5.0	–
	F_REG2_			10 × 10	0.250	0.20	5.0	
Random cuboids	F_RAND1_	1	1	2000 × 2000	0.050	0.0080	7.0	Span range of urban values in
	F_RAND2_				0.050	0.0081	25.0	Loridan and Grimmond (2012)
	F_RAND3_				0.50	0.057	7.0	
	F_RAND4_				0.50	0.057	25.0	
London low LOD	F_Lon, L_	1	1	2000 × 2000	0.4407	0.055	25.5	Building raster and footprints Google Inc. (2019), Evans et al. (2006)
London high LOD	F_Lon, H_	1	1	2000 × 2000	0.5095	0.075	19.6	As London low LOD
Indianapolis high LOD	F_Ind, H_	1	1	2000 × 2000	0.3461	0.037	17.9	Building raster from Google Inc. (2019) imagery and Heris et al. (2020) building footprints

The three real-world domains (i.e., 3 and 4) are 2000 × 2000 m^2^, to sample a wide range of streets with different widths, orientation, intersections, parking areas, plazas, and parks.

The DART model uses vector 3D building models to describe the urban form. For the low LOD, a raster digital surface model (DSM) and digital elevation model (DEM) are used to determine the building roof and ground level from the “Virtual London” building footprint dataset (Evans et al. 2006), using the 25th percentile of the DEM height, and the 75th percentile of the DSM height. Each building is assigned one height value from these building footprints. The 3D building models in the high LOD domains are created using the Morrison et al. (2020a, b) method from Google Earth imagery (Google Inc. 2019) and building footprints (Evans et al. 2006; Heris et al. 2020).

For SPARTACUS-Urban, profiles of *λ*_*p*_ and *L* are calculated from rasterized (1 m resolution) building heights derived from the 3D building models. As SPARTACUS-Urban assumes no topographic variation within an individual NWP grid cell (i.e., flat), the DART 3D array of voxels is re-gridded to give heights relative to local ground level for high LOD scenes (Morrison and Benjamin 2021). To balance computational time and simulation resolution, the DART voxel resolution used is 1 m vertically and 15 m horizontally for both the real-world and F_RAND_ scenes. For F_REG_, a vertical resolution of 0.5 m is used. In all scenes, SPARTACUS-Urban uses the same vertical resolution as DART.

As the real-world 3D building models are found to not conserve energy in DART, primarily because of periodic boundary conditions, the energy loss (always < 2%) needs to be redistributed. The rationale and method are explained in Appendix 2.

Fluxes from the DART model for F_REG_ (Table 1) scenes are offset by the voxel vertical resolution, as DART provides the fluxes at the ‘top face’ of each voxel and all roofs are at 5 m, so at the top of a voxel. As SPARTACUS-Urban outputs wall absorption profiles between height intervals, DART wall absorption for F_REG_ scenes is offset by 0.25 m. All data used and code are archived at https://zenodo.org/10.5281/zenodo.5145851.

### Sun Angles and Albedos

Both the DART and SPARTACUS-Urban models require solar zenith angle, *θ*_*0*_, to be provided for a simulation. For computational simplicity we use three: directly overhead (0°, although unrealistic when out of the tropics), 45°, and low-sun conditions (75°). Incoming radiation at the top of the canopy is assumed to be directly from the sun; diffuse incoming radiation is set to zero. Similarly, a material albedo (*α*) is needed. We use two values: low (0.1) as observed in dense urban areas (e.g., 0.11, Kotthaus and Grimmond 2014) and high (0.5) as typical of ‘cool’ materials (e.g., Santamouris 2014; Santamouris et al. 2018; Jandaghian and Akbari 2018).

As SPARTACUS-Urban assumes that the azimuthal orientation of buildings is random, solar azimuth angle (*Ω*) is not specified by the model, whereas for DART the value of *Ω* is specified. Thus, DART has varying shadow patterns with *Ω* used. The DART simulations use four values for the simpler F_RAND_ and F_REG_ cases, and eight (at 45° intervals) for the real-world cases. The final DART fluxes for comparison use the mean across all *Ω* intervals.

### Evaluation Statistics

To quantify SPARTACUS-Urban performance, we compare SPARTACUS-Urban and DART profiles of: mean shortwave upwelling (SW_↑_) and downwelling clear-air (SW_↓_) fluxes, and mean wall (*a*_Wall_) and roof shortwave absorption (*a*_Roof_). The fluxes have units of watts per square metre (W m^−2^) of the entire horizontal scene (rather than per square metre of the clear-air region excluding buildings), while the absorptions have units of W m^−3^, since we divide the absorption in a layer by the layer thickness to obtain a resolution-independent quantity. Thus, the vertical integral of *a*_Wall_ and *a*_Roof_ provide the total wall and roof absorptions (again per unit area of the entire horizontal scene). Unlike the vertical walls assumed by SPARTACUS-Urban for all domains, for the high LOD geometry in DART there is no simple way to distinguish or define roofs and walls. Hence, we combine the wall and roof absorption (*a*_Wall+Roof_) for the evaluation of the high LOD scenes. All fluxes and absorptions are normalized by the bottom of atmosphere (BOA) shortwave flux (SW_↓,BOA_). This is defined as the incoming shortwave flux across a horizontal plane above the tallest roughness elements in a scene (Gastellu-Etchegorry et al. 2015; Wang et al. 2020).

Profiles of *a*_Wall_ and *a*_Roof_ are compared using the normalized mean-absolute error (*nMAE*) and normalized mean-bias error (*nMBE*) expressed as a percentage of the mean DART absorption

12 $$ \begin{array}{*{20}c} {{nMAE} = \frac{{\frac{1}{n}\sum \left| {a_{{{\text{SU}}}} -a_{{{\text{DART}}}} } \right|}}{{\frac{1}{n}\sum a_{{{\text{DART}}}} }} 100 ,} \\ \end{array} $$

13 $$ \begin{array}{*{20}c} {{nMBE} = \frac{{\frac{1}{n}\sum \left( {a_{{{\text{SU}}}} -a_{{{\text{DART}}}} } \right)}}{{\frac{1}{n}\sum a_{{{\text{DART}}}} }} 100 ,} \\ \end{array} $$

where *a*_SU_ and *a*_DART_ are the flux at each height from SPARTACUS-Urban or DART, respectively. The metrics *nMAE* and *nMBE* are calculated at scene resolution (e.g., 1 m) vertically from 1 m to the maximum height in DART (*H*_*max*_). The SW_↑_ flux profiles are evaluated using the normalized bias error at a specified height (*nBE*), expressed as a percentage of the DART flux:

14 $$ \begin{array}{*{20}c} {{nBE} = \frac{{{{SW}}_{{{\text{SU}}}} -{{SW}}_{{{\text{DART}}}} }}{{{{SW}}_{{{\text{DART}}}} }}100 . } \\ \end{array} $$

The scene albedo is evaluated using SW_↑_ at the top of the canopy (*H*_max_ in DART). We also use the metric *nBE* to evaluate the total ground absorption (*a*_Ground_).

## Test of the SPARTACUS-Urban Geometry Assumptions

We examine the underpinning SPARTACUS-Urban assumption—that urban buildings are randomly distributed, or equivalently their horizontal separations follow an exponential distribution (Eq. 2)—by analyzing probability distributions from real cities and domains containing randomly placed cuboids (Table 1).

**Fig. 2:**
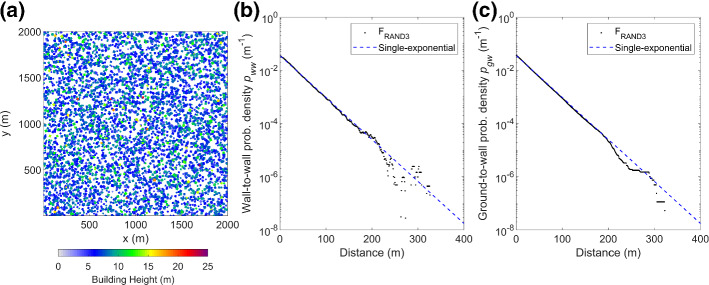
Randomly placed cuboid buildings (F_RAND3_, Table 1) within a 2 × 2 km^2^ domain with a plan area fraction at the surface, (*λ*_*p,0*_) of 0.5 and a mean height ($\overline{H }$) of 7 m: **a** Plan view, randomly placed cuboid buildings and probability density with a single-exponential (Eq. 2) fits to the **b** wall-to-wall and **c** ground-to-wall probability distributions

For the high density F_RAND3_ domain, the ‘true’ probability density of wall-to-wall (*p*_ww_) and ground-to-wall (*p*_gw_) separations (Fig. 2) are calculated following Hogan (2019b) with 1 × 1 m^2^ resolution building rasters, analyzed in four azimuthal directions 45° apart. Both the *p*_ww_ and *p*_gw_ distributions fit a single-exponential well (Fig. 2b, c) for separations up to 200 m, indicating F_RAND3_ satisfies SPARTACUS-Urban’s assumption of randomly distributed buildings. This behaviour is seen for all F_RAND_ domains. Here we use Eq. 2, where *X* is obtained from Eq. 5 with the surface value of *L* (denoted *L*_*0*_).

**Fig. 3:**
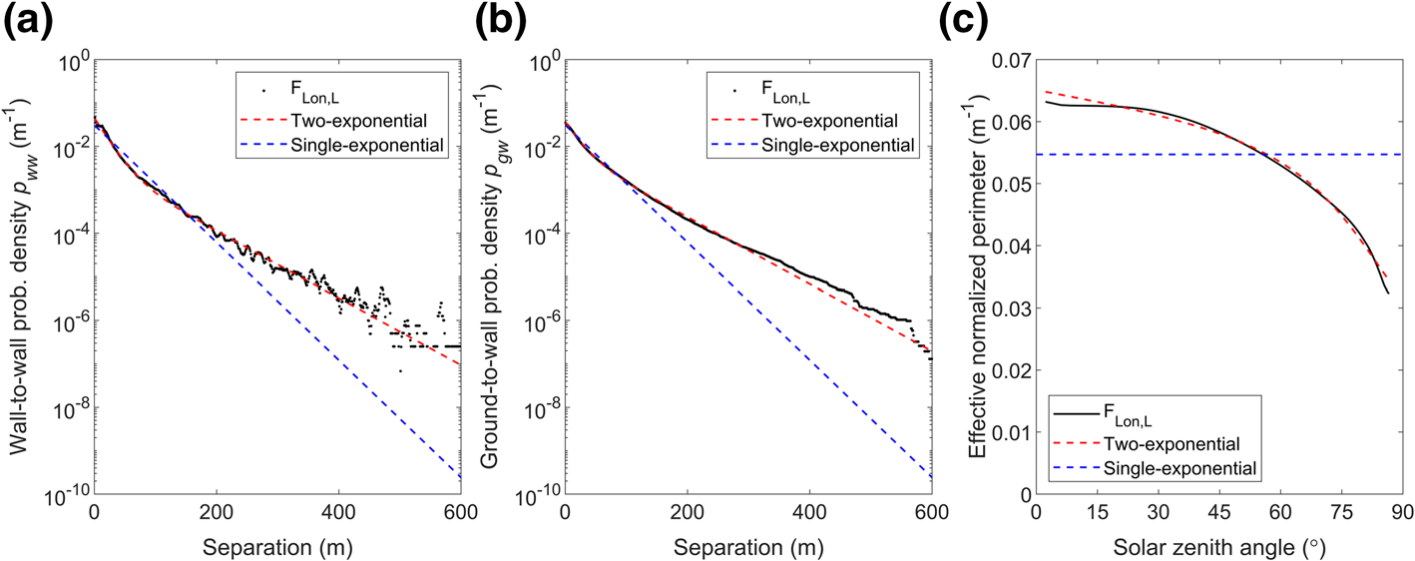
For a 2 × 2 km^2^ area of central London at low LOD (F_Lon,L_): **a** wall-to-wall and **b** ground-to-wall probability distribution, with single- (Eq. 2, blue) and two-exponential fit (Eq. 3 where *X*_1_ = 21.6 m, *X*_2_ = 57.4 m and *G*_*gw*_ = 0.534, red), and **c** corresponding effective normalized perimeter length at the surface as a function of solar zenith angle, ${\hat{L}_{0}}$(*θ*_0_)*,* as predicted by Eq. 9 when applied to the actual data (black) and fitted (red) ground-to-wall probability distributions, and the true perimeter length at the surface (blue)

**Fig. 4:**
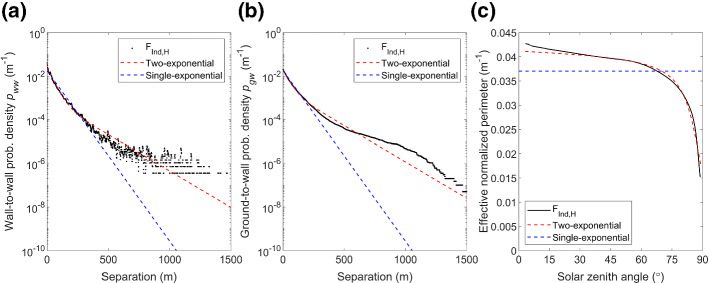
As Fig. 3, but for Indianapolis at high LOD (F_Ind,H_)

Figures 3 and 4 present similar analyses for London and Indianapolis respectively, although here eight azimuthal directions are used to determine *p*_gw_ and *p*_ww_, but offset by 22.5° to not align with major streets orientations (e.g., north–south or east–west in Indianapolis, Fig. 1c). Comparison of the calculated probability distribution to both the single- and two-exponential fits for central London indicates that the latter is a better fit for F_Lon,L_ (Fig. 3) and F_Lon,H_ (Online Resource 1). For F_Lon,L_, the single-exponential fit diverges from the *p*_*ww*_ distribution at approximately 200 m and from the *p*_*gw*_ distribution at approximately 100 m (Fig. 3b, c), whereas for F_Ind,H_ (Fig. 4) the single-exponential fits diverge at slightly greater distances (these numbers increasing to around 300 and 200 m, respectively). By contrast, the two-exponential (Eq. 3) predicts the larger building-separations much better than the single-exponential in both cities.

For F_Lon,L_ the effective normalized building perimeter length, ${\widehat{L}}_{0}$, decreases when *θ*_0_ > 30° (black line Fig. 3c). This is computed using Eq. 5 with the true ground-to-wall probability distribution (i.e., Fig. 3b), the mean building height ($\overline{H }$ = 25.5 m), and *F*_0g_ (Eq. 6). This shows that more direct solar radiation reaches the surface when the sun is low in the sky (i.e., less chance of wall interception) compared to purely randomly distributed buildings. Using the actual normalized perimeter of 0.055 m^−1^ (blue, Fig. 3c), equivalent to the single-exponential assumption, would be expected to lead to an overpredicted interception of direct solar radiation by walls for larger *θ*_0_. The $\widehat{L}$ value obtained from Eq. 9 with the two-exponential method agrees well with $\widehat{L}$ obtained using the true probability (Fig. 3c), and the same behaviour is seen for Indianapolis (Fig. 4c) ($\overline{H }$ = 17.9 m). Thus, we expect that the two-exponential fit should improve SPARTACUS-Urban simulations for real-world cities. This is tested in Sect. 6.

## Evaluation of SPARTACUS-Urban Shortwave Fluxes

### Regular Cubes

**Fig. 5:**
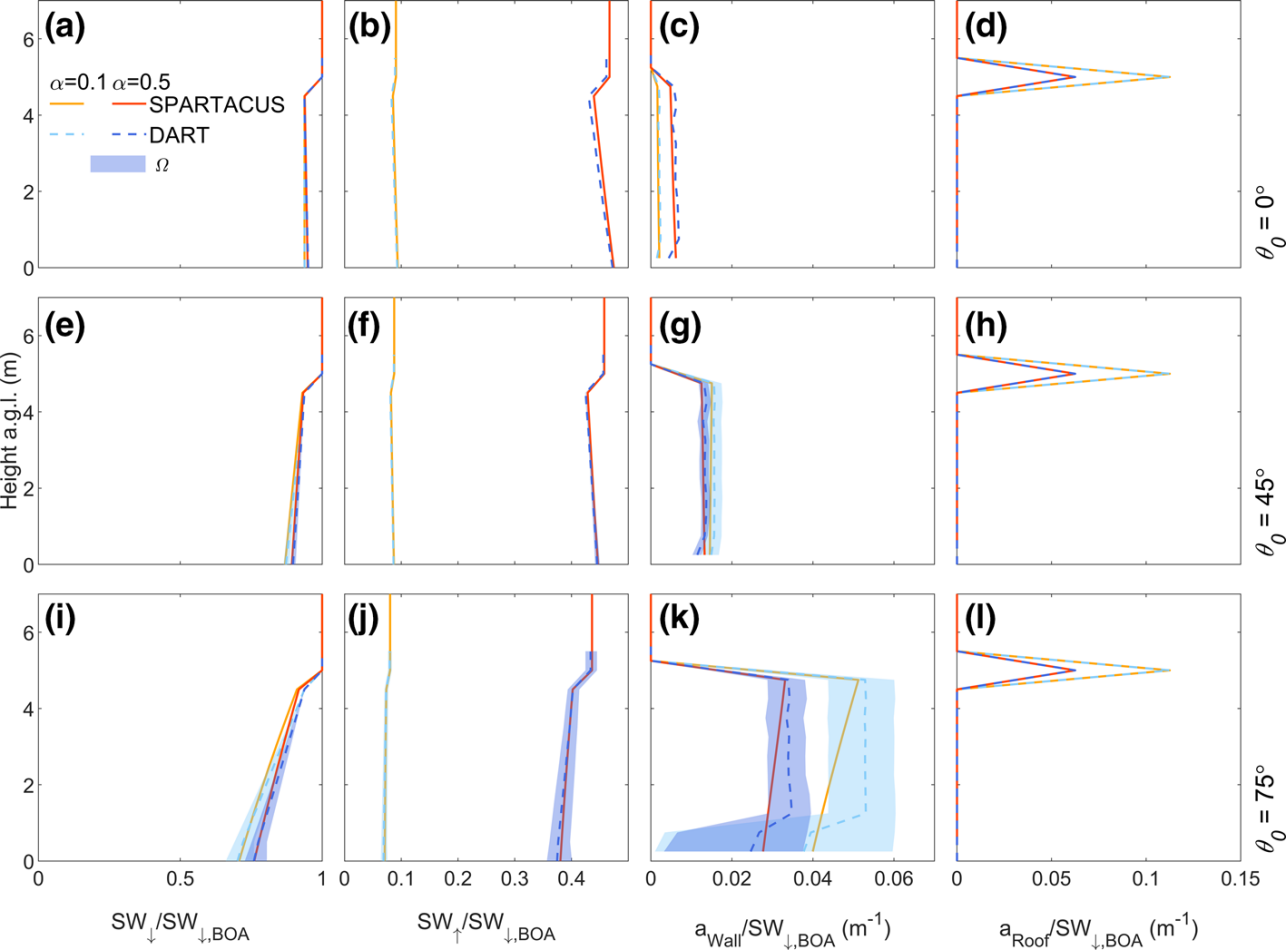
Fluxes for a regular repeated array of 5 × 5 × 5 m^3^ cubes (F_REG1,_ Table 1) normalized by the BOA flux (SW_↓,BOA_) with height, simulated with SPARTACUS-Urban (orange) and DART (blue), for two albedos (*α*: 0.1, 0.5) and three solar zenith angles (*θ*_*0*_: 0°, 45°, 75°): (**a**, **e**, **i**) downwelling clear air flux (SW_↓_), (**b**, **f**, **j**) upwelling clear air flux (SW_↑_), (**c**, **g**, **k**) wall absorption (*a*_Wall_), (**d**, **h**, **l**) roof absorption (*a*_Roof_) with solar azimuth angle (*Ω*) dependence in DART (shading)

Comparison of shortwave radiative flux profiles simulated with DART and SPARTACUS-Urban (single-exponential, Eq. 2) in a low-density regular array of cubes (F_REG1_) shows that SW_↓_ decreases closer to the surface when the zenith angle *θ*_*0*_ = 75° (Fig. 5) because more radiation is intercepted by buildings. Hence, less shortwave radiation penetrates to ground level. For all *θ*_*0*_ values, the roof absorption, *a*_Roof_, remains constant, with *nMAE* = 0 (i.e., machine precision). As the buildings are all the same height, *a*_Roof_ can be computed exactly from the building fraction and albedo. Maximum values of *a*_Wall_ increase with *θ*_*0*_ (Fig. 5c, g, k), with *a*_Wall_ increasing with height due to building shadowing at the surface (as *θ*_*0*_ increases). Azimuth angle (*Ω*) variations change shadow patterns and alter the wall area exposed to shortwave radiation. Hence, the *a*_Wall_ vertical profiles differ between DART and SPARTACUS-Urban. The DART model’s fluxes are averaged across four *Ω* values (Sect. 4.2). The SPARTACUS-Urban model’s *a*_Wall_ profiles are within the DART range arising from *Ω* (Fig. 5c, g, k shading) when *θ*_0_ = 0° and 45°, but not when *θ*_*0*_ = 75° and *α* = 0.1 near the surface (approximately 1 m). Errors in a_Wall_ are lowest when *θ*_*0*_ = 45° (*nMAE* between 9.9 and 17%, *nMBE* between −7.5 and 1.8%). For the scene albedo the *nBE* are < 1.2%, with values highest if the sun is overhead (*θ*_*0*_ = 0°). When *α* = 0.1, *nBE* in SW_↑_ and SW_↓_ increase, but are still < 2% (Online Resource 2). These results are better than expected, given the grid arrangement of the buildings do not have an exponential distribution of building separations (i.e. as SPARTACUS-Urban assumes, Sect. 2.1).

**Fig. 6:**
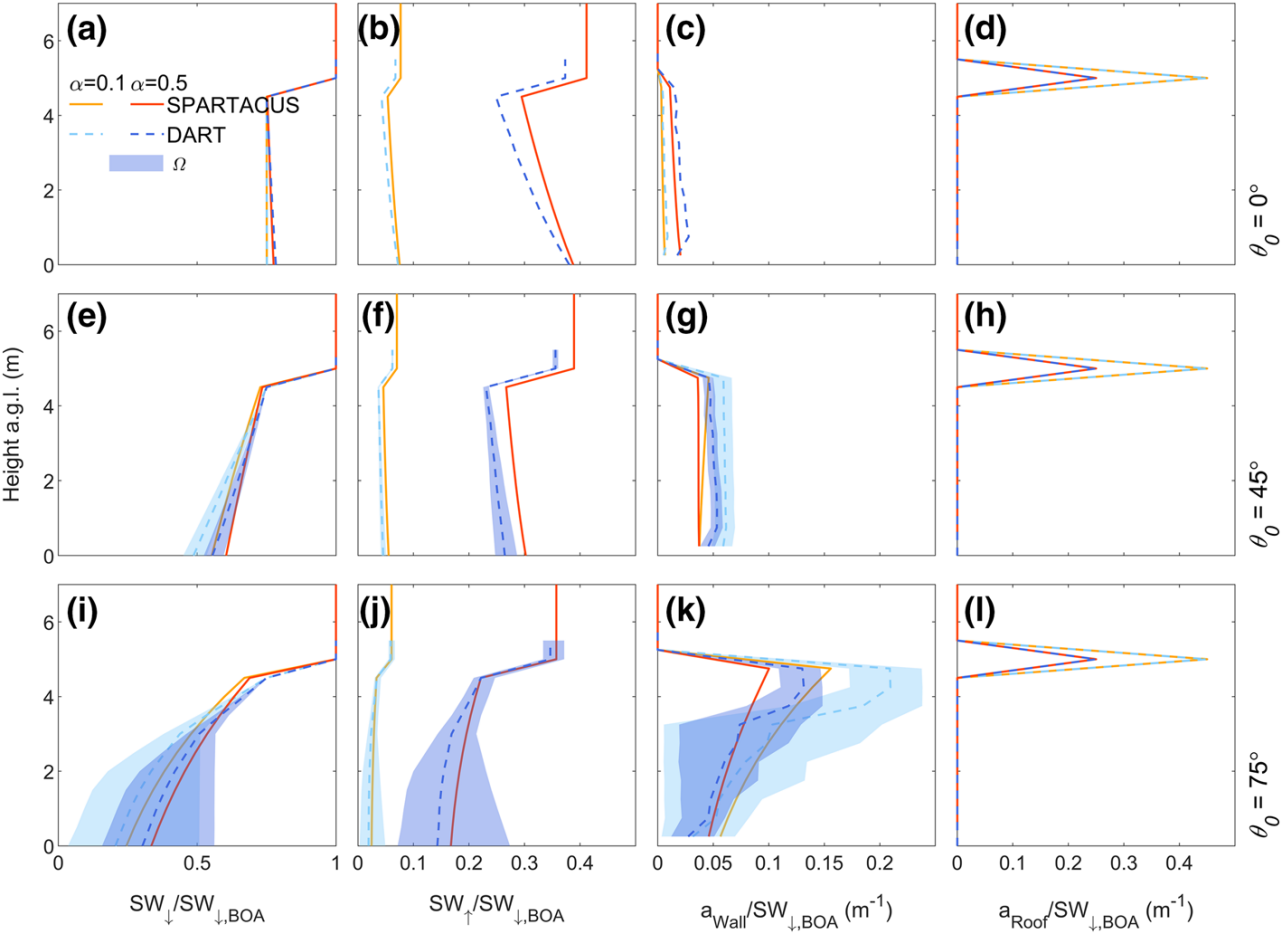
As Fig. 5, but for F_REG2_

The larger plan area index of F_REG2_ (Table 1) causes SW_↓_ to decrease more as height decreases (for high *θ*_*0*_) (Fig. 6a, e, i). The form F_REG2_ also increases mutual building shadowing, reducing the shortwave radiation penetrating to the surface. With less shortwave radiation escaping, the scene albedo decreases with increasing *θ*_*0*_. The metric *nBE* is larger (up to 10%, Table 2b) cf. F_REG1_. Maximum values of *a*_Wall_ increase as *θ*_*0*_ increases (Fig. 6c, g, k). However, *a*_Wall_ decreases more rapidly as height decreases than in F_REG1_, due to the increased shadowing from the buildings/cubes. The value of * nMAE* is larger (cf. F_REG1_) for *a*_Wall_ (up to 35%, Table 2b). The peak in *a*_Roof_ remains at 5 m, as all buildings are of equal height. The SPARTACUS-Urban model generally overpredicts the SW_↑_ and SW_↓_ profiles, and underestimates *a*_Wall_ at the top of the canopy (Fig. 6) in F_REG2_. Similarly to F_REG1_, when *α* = 0.1 *nMAE* in *a*_Wall_ can be up to 35% when *θ*_*0*_ > 45° (Online Resource 2).

**Table 2:** Evaluation of SPARTACUS-Urban (relative to DART) for F_REG_ scenes (Table 1) (a) F_REG1_ and (b) F_REG2_, for three solar zenith angles (*θ*_*0*_: 0, 45, 75°) and one albedo (*α*: 0.5). Upwelling and downwelling clear air shortwave flux profiles (SW_↑_ and SW_↓_) assessed with the normalized bias error (nBE, Eq. 14) and wall and roof absorption (*a*_Wall_, *a*_Roof_) profiles assessed with the normalized mean absolute error (*nMAE*, Eq. 12) and the normalized mean-bias error (*nMBE*, Eq. 13)

*θ*_0_ (°)	Scene Albedo (*z* = 5.5 m)		*a*_Ground_		*a*_Wall_	
	DART	*nBE* (%)	DART	*nBE* (%)	*nMAE* (%)	*nMBE* (%)
*(a) F*_*REG1*_						
0	0.461	1.2	0.476	−0.25	17	−7.5
45	0.456	0.36	0.446	0.13	9.9	1.8
75	0.434	0.57	0.372	2.2	16	−3.8
*(b) F*_*REG2*_						
0	0.373	10	0.392	−1.2	29	−22
45	0.356	9.4	0.264	14	31	−22
75	0.346	3.1	0.142	18	26	−8.3

### Random Cubes

Four F_RAND_ domains (F_RAND1_ to F_RAND4_) are intended to test SPARTACUS-Urban performance across the *λ*_*p*,0_ and $\overline{H }$ extreme combinations, with more results for eight other F_RAND_ domains given in Online Resource 3. All F_RAND_ simulations use the single-exponential model (Eq. 2), as it fits the building distribution data well (Fig. 2).

**Fig. 7:**
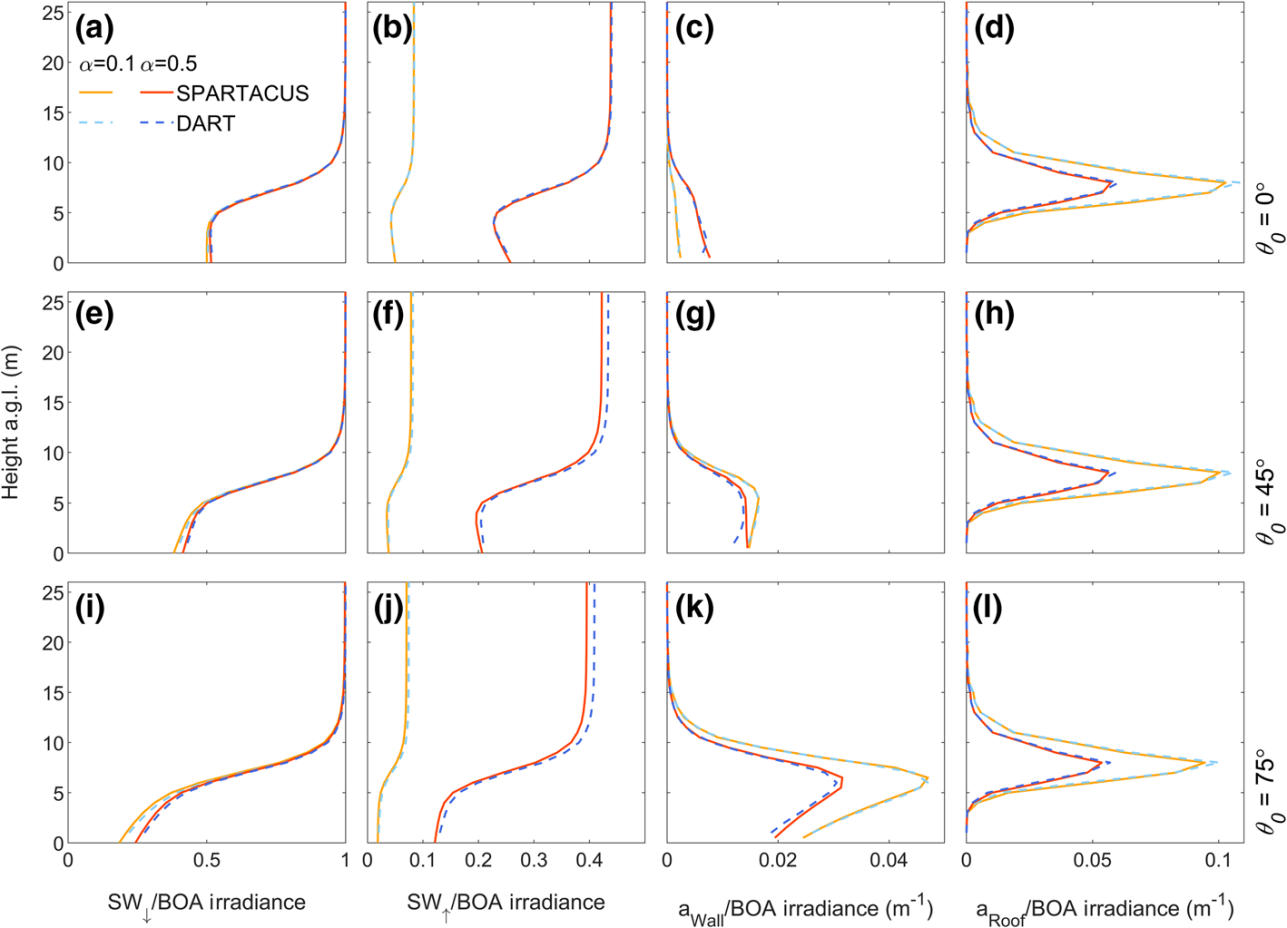
As Fig. 5, but for F_RAND3_

Figure 7 shows the agreement between DART and SPARTACUS-Urban for each *θ*_*0*_ and *α* for profiles of *a*_Wall_, *a*_Roof_, and SW_↓_ for F_RAND3_. Overall, for F_RAND1_–F_RAND4_, the *nBE* and *nMAE* are less than 6% for all quantities (Table 3), as SPARTACUS-Urban’s urban form assumptions (Sect. 5) are fulfilled. The SPARTACUS-Urban model agrees better with DART when *λ*_*p*,0_ and $\overline{H }$ are small, as buildings are further apart so there is less within-canyon scattering and building shadowing. The largest differences between DART and SPARTACUS-Urban are seen for *a*_Wall_ between 1 and 5 m for *θ*_*0*_ = 45°, 75°. The SPARTACUS-Urban model underestimates SW_↑_ for *θ*_*0*_ = 0° and 45°. In F_RAND1-2_, when *λ*_*p*,0_ = 0.05, *nBE* < 0.7% compared to *nBE* = 3.4–5.0% when *λ*_*p*,0_ = 0.5 (F_RAND3-4_). When *λ*_*p*,0_ = 0.05, *nMAE* < 1%, except for *a*_Wall_ in F_RAND1_, where nMAE = 2.1%. Although performance becomes poorer as *λ*_*p*,0_ increases, F_RAND3-4_ errors do not exceed 5.5% when *θ*_*0*_ = 75° and *α* = 0.5. The differences in *nBE* and *nMAE* magnitudes are larger with an increase in *λ*_*p*,0_ (F_RAND1_–F_RAND3_), compared with an increase in $\overline{H }$ (F_RAND1_–F_RAND2_). This is also seen in the additional scenes in Online Resource 3.

**Table 3:** As Table 2, but for F_RAND_ (Table 1) with *θ*_*0*_ = 75°. *H*_max_ is the maximum building height in DART. Additional cases are given in Online Resource 3

F_RAND_	*H*_max_ (m)	Scene Albedo (SW_↑_, *z* = *H*_max_)		*a*_Ground_		*a*_Wall_		*a*_Roof_	
		DART	*nBE* (%)	DART	*nBE* (%)	nMAE (%)	*nMBE* (%)	*nMAE* (%)	*nMBE* (%)
1	17	0.483	−0.65	0.451	−0.51	2.1	1.8	1.0	0.68
2	75	0.443	−0.71	0.394	−0.55	2.5	2.3	3.5	0.59
3	26	0.409	−3.4	0.126	−3.4	4.3	4.3	5.1	−0.11
4	81	0.333	−4.7	0.0358	−5.0	5.5	4.7	5.5	−0.61

### Real-World Geometry

For the real-world urban form in SPARTACUS-Urban, both the single- (Eq. 2) and two-exponential (Eq. 3) fits are used, allowing assessment of the impact of the building layout assumptions on shortwave radiative fluxes. Although errors are discussed here, the range of *C* values in real-world cities (Appendix 1) means that the SPARTACUS-Urban flux calculations could be adjusted based on the exact value of *C* used.

**Fig. 8:**
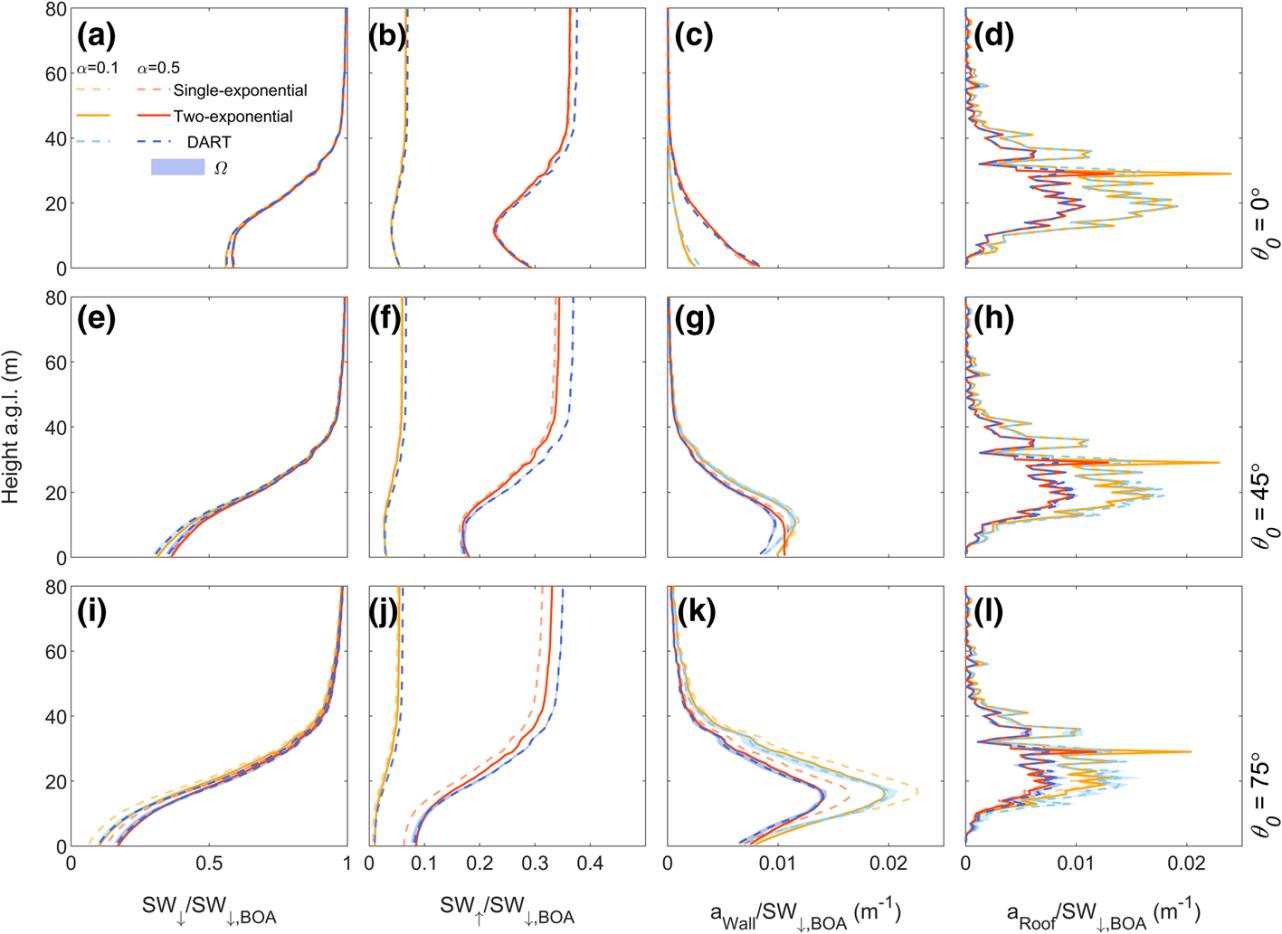
As Fig. 5, but for London with low LOD (F_Lon, *L*_) and SPARTACUS-Urban using single- (dashed) and two-exponential (solid) fits

Building height distribution profiles have spikier patterns for F_Lon,L_ than F_Lon,H_ (blue and orange, Online Resource 4c), with the largest differences between 25 and 50 m. These spikes occur because individual buildings in the F_Lon,L_ domain each have only one height, and are aggregated per 1 m interval. Despite this, the vertical profiles of *a*_Roof_ in SPARTACUS-Urban and DART are still close (Fig. 8d, h, l).

The SPARTACUS-Urban model has good agreement to DART for F_Lon,L_ vertical flux profiles (Fig. 8), although the agreement is generally poorer with increasing *θ*_*0*_. The SPARTACUS-Urban model always underestimates SW_↑_, with *nBE* values within 7% of DART (Table 4a). In the SPARTACUS-Urban model, *a*_Ground_ is overestimated but with *nBE* < 6%. The SPARTACUS-Urban model is generally in better agreement to the DART model for both SW_↑_ and *a*_Ground_ when using the two-exponential method_._ This is most evident as *θ*_*0*_ increases. Neither *nMAE* nor *nMBE* exceed 7.3% for *a*_Wall_. Generally, the SPARTACUS-Urban model underestimates *a*_Roof_, with *nMAE* < 12%, and *nMBE* up to −4.3% (increases to 13% and −6.3% for the single-exponential method). When *α* = 0.1 for the two-exponential, the maximum magnitudes of nBE for SW_↑_ increases (10%, Online Resource 5) but *nBE* for *a*_Ground_, and *nMAE* for *a*_Roof_ are similar (cf. *α* = 0.5).

**Table 4:** As Table 2, but for (a) F_Lon, L_, (b) F_Lon,H_, and (c) F_Ind,H_ using the single (Eq. 2) and two-exponential (Eq. 3) fit methods for urban geometry, with albedo = 0.5

*θ*_0_ (°)	Scene Albedo (*z* = *H*_max_)			*a*_Ground_			*a*_Wall_				*a*_Roof_			
	DART	Single	Two	DART	Single	Two	Single	Two	Single	Two	Single	Two	Single	Two
		*nBE* (%)			*nBE* (%)		*nMAE* (%)		*nMBE* (%)		*nMAE* (%)		*nMBE* (%)	
*(a) F*_*Lon,L*_														
0	0.378	−0.70	−3.4	0.293	0.18	0.41	10	6.7	−3.8	4.3	7.8	7.8	1.3	1.3
45	0.372	−6.3	−6.9	0.171	7.4	6.0	5.2	7.3	4.9	6.7	9.3	9.4	−3.1	−3.2
75	0.355	−8.6	−5.5	0.0796	−13	6.2	13	4.9	13	4.8	13	12	−6.3	−4.3

*θ*_*0*_ (°)	Scene Albedo (*z* = *H*_max_)			*a*_Ground_			*a*_Wall+Roof_			
	DART	Single	Two	DART	Single	Two	Single		Two	
		*nBE* (%)			*nBE* (%)		*nMAE* (%)	*nMBE* (%)	*nMAE* (%)	*nMBE* (%)
*(b) F*_*Lon,H*_										
0	0.360	3.7	1.6	0.254	−10	−9.8	6.6	2.7	6.8	4.3
45	0.353	−1.9	−2.5	0.172	−21	−22	9.0	8.2	9.3	8.9
75	0.342	−5.9	−3.4	0.0794	−39	−27	12	8.4	12	5.3
*(c) F*_*Ind,H*_										
0	0.426	1.2	0.32	0.351	−4.6	−4.5	11	4.9	11	6.3
45	0.420	−1.7	−2.4	0.310	−8.5	−9.1	13	12	14	14
75	0.394	−4.2	−3.7	0.226	−17	−16	15	14	15	13

**Fig. 9:**
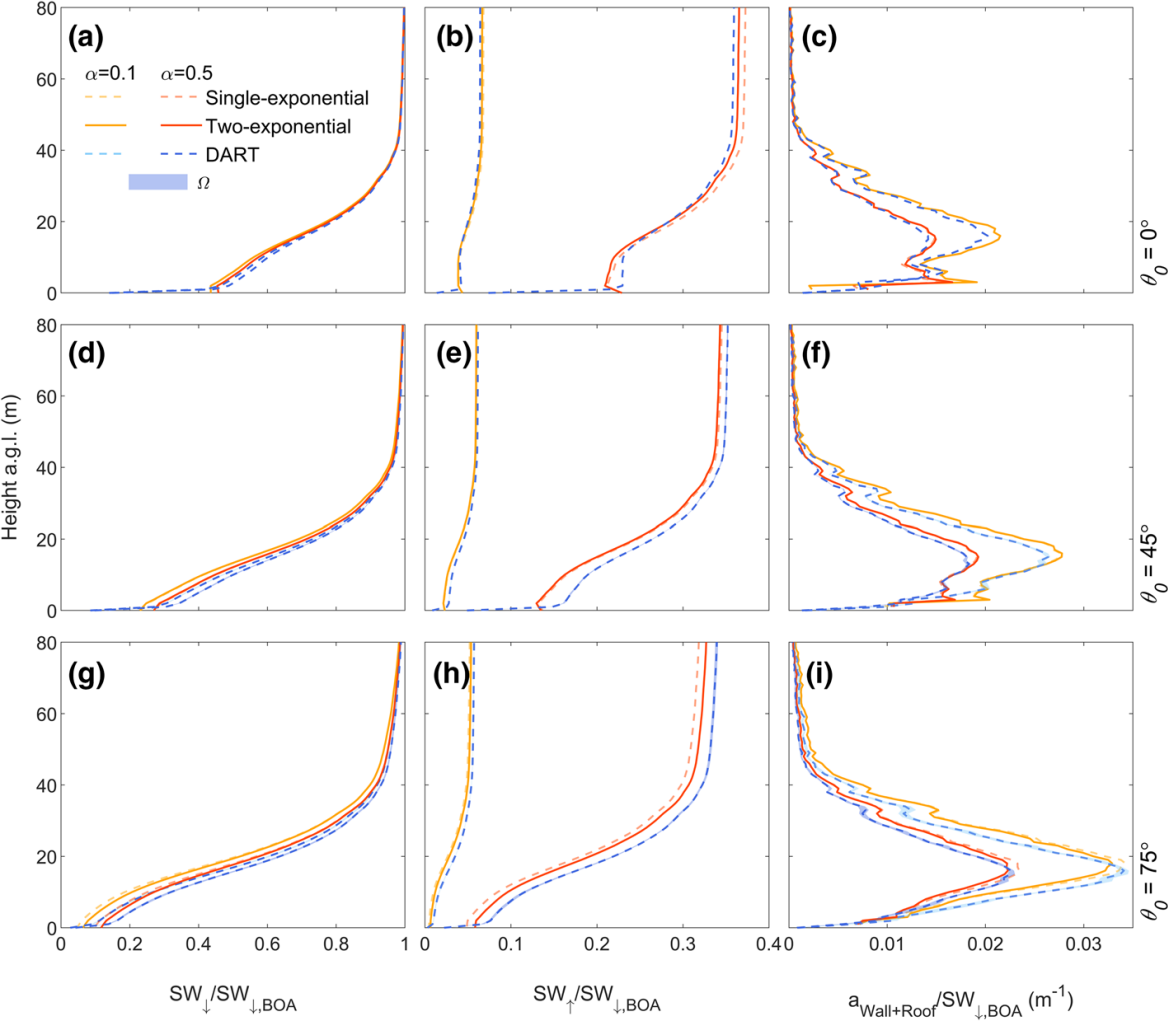
Fluxes normalized by the BOA flux (SW_↓,BOA_) for a high LOD 2 × 2 km^2^ domain in central London (F_Lon, H_, Table 1), using SPARTACUS-Urban (orange) and DART (blue) for two albedos (*α*: 0.1 and 0.5) and three solar zenith angles (*θ*_0_*:* 0°, 45°, 75°) with solar azimuth angle (*Ω*) variation in DART (shading): (**a**, **d**, **g**) downwelling clear air flux (SW_↓_), (**b**, **e**, **h**) upwelling clear air flux (SW_↑_), (**c**, **f**, **i**) wall-plus-roof absorption (*a*_Wall+Roof_). The SPARTACUS-Urban model’s radiative fluxes are computed using both single (Eq. 2, orange solid) and two- (Eq. 3, orange dashed) exponential fits

The vertical absorption profiles (*a*_Wall_, *a*_Roof_, *a*_Wall+Roof_) for the London scenes are well captured by SPARTACUS-Urban (Figs. 8 and 9). The *a*_Wall+Roof_ maxima between DART and SPARTACUS-Urban (Fig. 9f, i) disagree mainly because of the need to adjust for intra-scene local topography heights in DART, in contrast to SPARTACUS-Urban where the ground is assumed to be flat. The SPARTACUS-Urban model fluxes for F_Lon,H_ are within 12% of DART, except for *a*_Ground_ when *θ*_0_ = 45° and 75° (*nBE* = −22%, −27%, Table 4b). Both scene albedo and transmission to the surface (Table 4b) are underestimated at all *θ*_*0*_. SPARTACUS-Urban overestimates the *a*_Wall+Roof_ profiles (Fig. 9c, f, i) leading to less shortwave radiation at ground level (with reduced SW_↓_ with decreasing height as *θ*_*0*_ increases). This leads to less shortwave reflected to the top of the canopy, reducing the scene albedo. The largest differences between the single- and two-exponential results occurs when *θ*_*0*_ = 75° (Table 4b). Simulations for F_Lon,H_ using *α* = 0.1 (cf. 0.5) have higher nBE magnitudes for both SW_↑_ (3.0–5.7%), and *a*_Ground_ (7.5–36%, Online Resource 5). Error metrics (*nBE*, *nMAE*, *nMBE*) are notably larger when using the single-exponential.

**Fig. 10:**
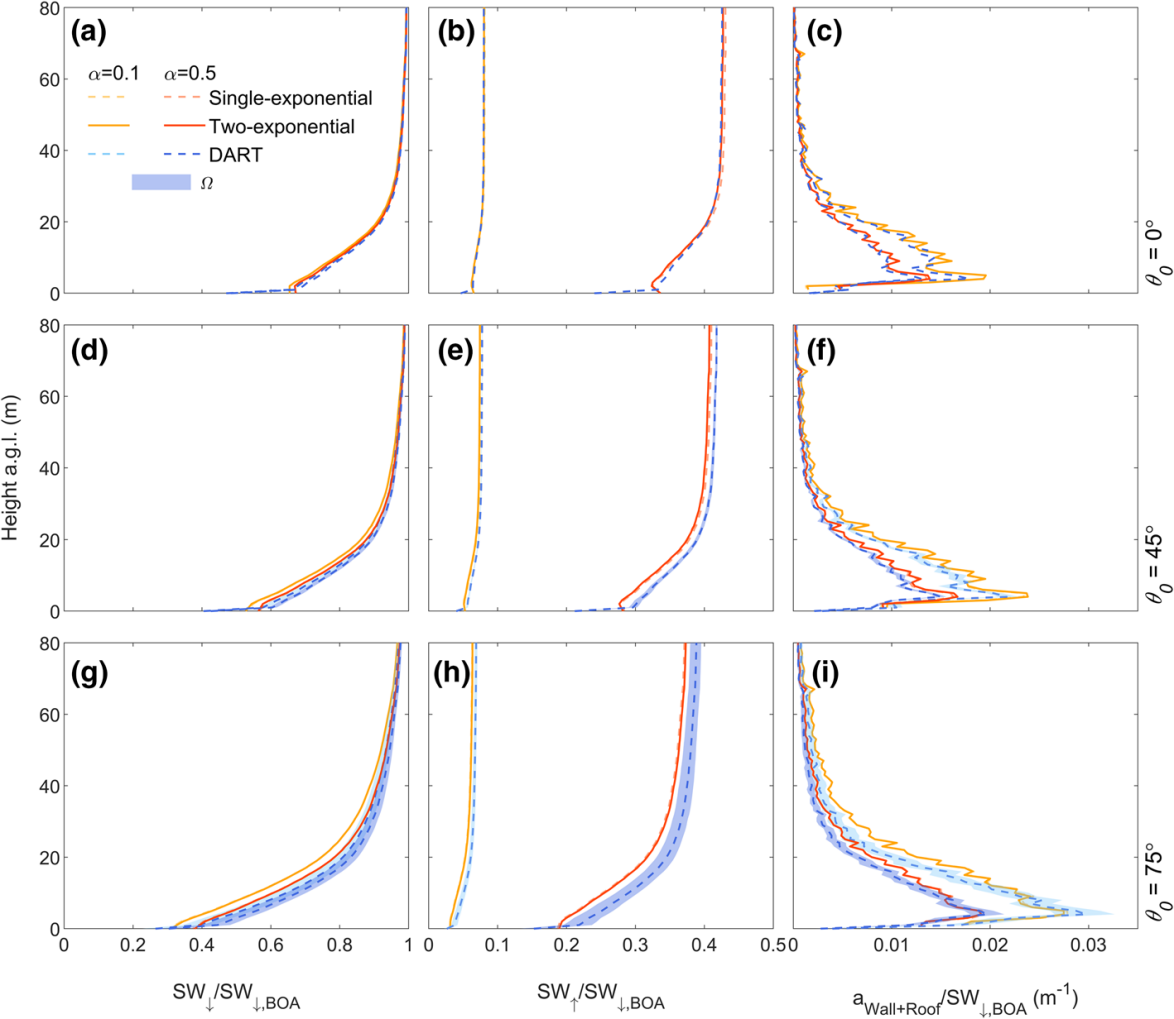
As Fig. 9, but for Indianapolis with a high level of detail (F_Ind,*H*_)

Both F_Ind,H_ SW_↑_ and *a*_Ground_ (hence SW_↓_) are generally underestimated, with *nBE* increasing with *θ*_0_ (0.32 to −3.7%, and −4.5 to −16% respectively, Table 4c). The SPARTACUS-Urban model overestimates *a*_Wall+Roof_ for all *θ*_0_ and *α* (Fig. 10c, f, i), with *nMAE* and *nMBE* between 6.3 and 15% (Table 4c) when using the two-exponential, with similar errors using the single-exponential. Using *α* = 0.1 increases the *nBE* in both SW_↑_ and *a*_Ground_ to −7% and −21% respectively (Online Resource 5).

## Comparison to the Single-Layer Infinite-Street-Canyon Assumption

Given the current urban models within NWP models commonly assume an urban form consisting of an infinite canyon with buildings of the same height and flat roofs, we assess SPARTACUS-Urban relative to one model of this type, Harman et al. (2004). This solves a small system of linear equations to treat any number of reflections within the canyon. Previously, Hogan (2019a) compared SPARTACUS-Urban to the Harman et al. (2004) longwave radiation by modifying the configuration, so assumptions are met for both, viz*.*: buildings all the same height, and exponential model of urban geometry (Hogan 2019b). Hogan (2019a) found excellent agreement between SPARTACUS-Urban using eight streams, supporting the use of the discrete ordinate method for urban radiative transfer.

Here, for shortwave radiation, we compare SPARTACUS-Urban and Harman et al. (2004) against DART for cases when the assumptions of the two models are not necessarily satisfied. The Harman method is used with its usual configuration (i.e., exchange coefficients consistent in a single-layer infinite street canyon as in Sect. 3 of Hogan (2019b)), and we implement the 2 × 2 matrix inversion approach of Harman et al. (2004), as outlined in Sect. 4.2 and Eq. 4 of Hogan (2019a). This approach assumes two parallel infinite length buildings have constant height, *H,* separated by street of constant width, *W*, with a fixed *H/W*.

Care is taken to ensure that in all comparisons, the total area of ground, wall, and roof is equal between the three models. For the Harman simulations, the height *H* is set equal to $\overline{H }$ (Table 1), and the building fraction equal to *λ*_*p,0*_. The value of *H/W* is calculated using the operational method in Eq. 3 of Hertwig et al. (2020),

15 $$ \begin{array}{*{20}c} {\frac{H}{W} = \frac{\pi }{2}\frac{{\lambda_{f} }}{{\left( {1 -\lambda_{p} } \right)}},} \\ \end{array} $$

where *λ*_*f*_ is calculated for each domain using Eq. 1 with the true wall area of the domain calculated from Δ*z* and *L*(*z*). From Eq. 15 we obtain *W* using $\overline{H }$. For SPARTACUS-Urban, we use the two-exponential form. Analysis is undertaken for both random cuboid and real-world scenes.

Unlike SPARTACUS-Urban, the Harman et al. method cannot predict vertical profiles, so the comparison of wall and roof absorptions is limited to vertically integrated quantities. Values of SW_↑_ at the top of the canopy are calculated for the *H*_max_ in each scene for DART and SPARTACUS-Urban. These are compared using *nBE* (Eq. 14). We expand on results for an albedo of 0.5 here, with results of the comparison for an albedo of 0.1 in Online Resource 6. Run times for five SPARTACUS-Urban configurations are compared. These have varying numbers of diffuse streams per hemisphere (*N* = 1 to 8) and layers: *n* = 1 (i.e., as Harman et al. (2004)) to 6 (e.g., reasonable for operational NWP), to 151 (i.e., this work). The computer time of a single-threaded run for a SPARTACUS-Urban profile with the most basic configuration (*N* = 1, *n* = 1) is fast (12 μs) but six times longer than for the Harman model (Table 5). The F_Lon,L_ scenes (Sect. 6.3; SPARTACUS-Urban configuration: *N* = 8, *n* = 151) have a much longer run time (9.2 ms) but SPARTACUS-Urban is ~ 2.5 million times faster than DART despite DART using 14 parallel threads (Table 5).

**Table 5:** Absolute run-time of the three models (Harman, SPARTACUS-Urban, and DART) for the low LOD London domain (F_Lon,L_, Table 1) with the indicated number of layers (*n*) and (for SPARTACUS-Urban) diffuse streams per hemisphere (*N*)

Model	*n*	*N*	Time (s)
Harman	1	–	1.8 × 10^–6^
SPARTACUS-Urban	1	1	1.2 × 10^–5^
	6	1	5.0 × 10^–5^
	151	1	1.1 × 10^–3^
	151	4	3.0 × 10^–3^
	151	8	9.2 × 10^–3^
DART	151	–	2.5 × 10^4^

The versions of SPARTACUS-Urban and Harman compared are both within the open-source SPARTACUS-Surface version 0.7.3 compiled with gfortran (O3 optimization). The runs are undertaken in a single-threaded Linux environment on a dual Xeon E5-2667 v3 processor with 256 GB of RAM. DART version 5.7.5 build number 1126 is run in the same Linux environment with 14 parallel threads using 32 CPU

**Fig. 11:**
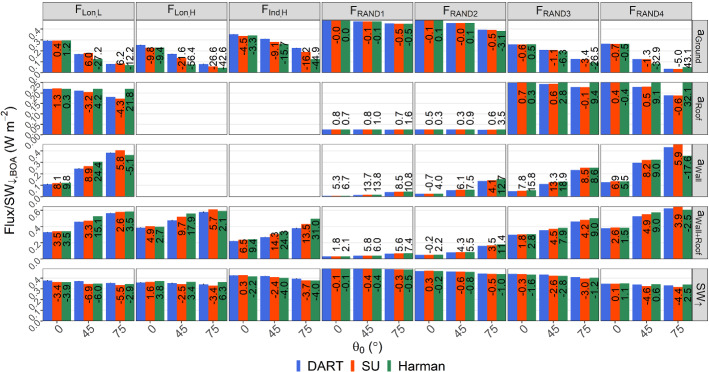
Comparison of SPARTACUS-Urban, Harman et al. (2004) and DART values for real-world scenes at low and high LOD (F_Lon, *L*_, F_Lon,*H*_, F_Ind,*H*_), and random cuboid scenes (F_RAND_) for three solar zenith angles (*θ*_0_*:* 0°, 45°, 75°) and an albedo of 0.5: upwelling clear air flux at the top of the canopy (SW_↑_), and total wall, roof, and ground absorption (*a*_Wall_, *a*_Roof_, *a*_Ground_). For high LOD scenes *a*_Wall_ and *a*_Roof_ are combined (*a*_Wall+Roof_) for evaluation. Numbers on each bar are the *nBE* (Eq. 14) between the SPARTACUS-Urban model/Harman approach and the DART model. Error bars for DART span the range between if no correction for the energy imbalance is made, and if double this correction is made (Appendix 2)

**Fig. 12:**
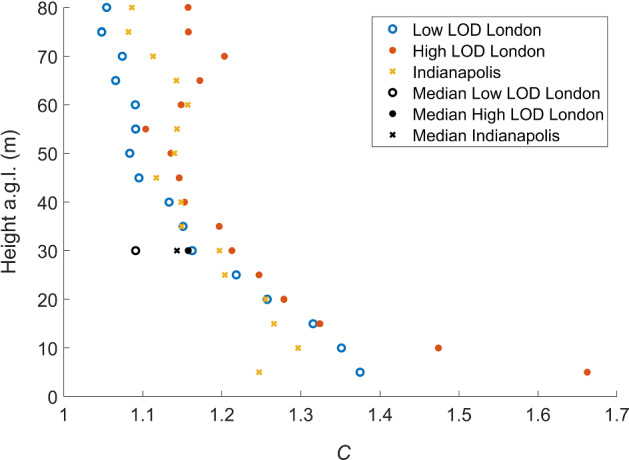
Variation of the concavity parameter (*C*) (Sect. 2.2) in two real cities (London and Indianapolis) for two LOD (Sect. 4.1) with height. The median values of *C* above $\overline{H }$ for each of the three domains, used as the scaling factor the building edge length (*L*) are shown in black

Overall, Harman et al. (2004) has the best agreement with DART SW_↑_ (*nBE* < 6.3%, Fig. 1) but generally overestimates *a*_Wall_, *a*_Roof_, and *a*_Wall+Roof_. The best agreement between Harman et al. (2004) and DART is found for F_RAND_ scenes (Fig. 11), however, SPARTACUS-Urban performs better (cf. Harman). Harman absorption errors increase with *θ*_*0*_, with *nBE* up to 18% or 32% (*a*_Wall_ and *a*_Roof_ respectively), compared with *nBE* for SPARTACUS-Urban (up to 13.7% or 0.8%).

For real-world domains, SPARTACUS-Urban performs better than the Harman approach, particularly for low *θ*_0_ (Fig. 11). The *nBE*_*SPARTACUS*_ values are in the range 1.3–14.3% for *a*_Roof_, *a*_Wall_, and *a*_Wall+Roof_, while *nBE*_*Harman*_ is 0.3–31% for the same quantities. The value of *a*_Roof_ is overestimated by both models in all cases, except for the SPARTACUS-Urban model for F_Lon,L_ at 45° and 75° (magnitudes of *nBE*_*SPARTACUS*_ < 4.3%, Fig. 11, cf. *nBE*_*Harman*_ < 21.8%), which is expected for the Harman approach as roof shadowing is neglected. Both models predict SW_↑_ well (*nBE* all < 7%), but the SPARTACUS-Urban model almost always performs better (Fig. 11). The worst performance for both models is for *a*_Ground_, with both models generally underestimating it (*nBE* of 0.4–26.6% for SPARTACUS-Urban and 1.2–56.4% for the Harman method). Overall, using SPARTACUS-Urban has a smaller *nBE* (cf. Harman) when evaluated using DART. For scenes where *nBE*_*Harman*_ (cf. DART) are lower than *nBE*_*SPARTACUS*_ (i.e., better performance), the differences in *nBE* are < 5%.

## Conclusions

Evaluation of the multi-layer SPARTACUS-Urban shortwave fluxes is undertaken using reference calculations from the explicit 3D radiative transfer model DART. The SPARTACUS-Urban model computes the vertical profiles of fluxes and absorption rates in urban scenes, which is crucial for vertically resolved urban energy balance models. A range of urban geometries were considered: regular arrays of cubes, cuboids with random placement and heights, to real city complexity (London and Indianapolis).

The SPARTACUS-Urban approach performs well when the SPARTACUS-Urban assumption of randomly distributed buildings is fulfilled. This is particularly evident for low building densities (*λ*_*p,0*_ = 0.05) where the normalized bias error (*nBE*) and normalized mean absolute error (*nMAE*) < 1%. The SPARTACUS-Urban model and the DART model agree less well as building fraction increases (*λ*_*p,0*_ = 0.5, *nBE* and *nMAE* < 5.5%). The largest *nBE* and *nMAE* occur when the solar zenith angle is highest (*θ*_*0*_ = 75°). For all random cuboid scenes presented, all *nBE* and *nMAE* are below 6%.

The shortwave radiative fluxes for real cities (London and Indianapolis) have *nBE* magnitudes of less than 7% for effective scene albedo, and *nBE* generally less than 15% for ground absorption. Exceptions to the latter occur for the high level of detail London domain when *θ*_0_ = 45° and 75°. Errors (*nMAE*) for the wall and roof absorption (low LOD) are less than 7% and 12%, respectively. The combined wall and roof absorption (high LOD domains) is always overestimated by SPARTACUS-Urban (*nMAE* < 15%), which leads to underestimation in the effective albedo of the scene, and underestimation in the transmission of shortwave radiation at the surface. However, the structure of the vertical profiles of fluxes and absorptions are captured well by SPARTACUS-Urban. Overall, upwelling profiles are best predicted by SPARTACUS-Urban. For the low LOD London domain, shortwave downwelling is typically overestimated, and roof absorptions are generally underestimated, in contrast to the high LOD domains. The performance of SPARTACUS-Urban in Indianapolis is slightly worse than for London scenes, which could be related to the grid-like street layout being further from the random building distribution assumed by SPARTACUS-Urban. Nonetheless, the Indianapolis domain used here still contains parks and diagonally oriented streets, which make the domain and building separations sufficiently random enough that SPARTACUS-Urban still performs well.

Regular cube arrays tested have a similar form to earlier urban radiation studies. The roof absorptions modelled in SPARTACUS-Urban are exact (cf. DART), as all buildings have the same height. The smallest differences between SPARTACUS-Urban and DART are found in scene albedo and ground absorption, where *nBE* < 2.2%. This increases to < 18% for denser cube arrays. Across all scenes, the largest differences between DART and SPARTACUS-Urban are found in wall absorption, with *nMBE* between 1.8 and −22% (*nMAE* 9.9% and 31%) depending on cube density. These errors in wall absorption are greater than in the real scenes, which given the regular spacing does not meet the randomly distributed buildings SPARTACUS-Urban assumption, this result is not unexpected. It is plausible the low *nMAE* and *nBE* results may be associated with the open cube spacing reduces building shadowing effects.

A modification to the original SPARTACUS-Urban method is introduced here, relaxing the strict assumption that the distribution of building separations fits a single-exponential (Eq. 2). This is replaced with a two-exponential method, allowing for effective building edge length to vary with *θ*_*0*_ (Eq. 3). This new method is proposed because Eq. 2 underpredicts the frequency of large building separations, and thus underpredicts the fraction of solar radiation reaching ground level. Using the two-exponential method both improves the predicted probability distributions (cf. ‘true’ distributions for London and Indianapolis) and reduces the SPARTACUS-Urban model’s radiative flux errors by up to a factor of a half. A further correction is applied to both the single- and two-exponential methods to account for the concavity of real buildings, leading to better representation of the width of building shadows. The range of possible concavity values in real-world cities means that SPARTACUS-Urban simulations could be calibrated to fit DART simulations. For NWP, the concavity value uncertainty for an individual domain is smaller than the uncertainty in *L*(*z*) itself.

There is scope to further refine SPARTACUS-Urban, including adjusting building shadowing. As SPARTACUS-Urban assumes the shadow cast by a building falls onto neighbouring buildings, or on gaps between buildings, is proportional to the roof area, this means shadows are randomly overlapped with roofs that they fall on. However, buildings often have roofs at low and high heights that are effectively next to each other when viewed from overhead. So, higher parts of a building can shadow lower roofs, rather than the street-level. Correcting this could improve the SPARTACUS-Urban performance further.

Comparison to the Harman et al. (2004) single-layer infinite street canyon model for randomly distributed cuboid, and real-world geometries found it performs best (c.f. DART) for random cuboid scenes. However, SPARTACUS-Urban generally performs better and notably in real-world scenes. The model results are most similar in their effective scene albedo predictions (*nBE* generally < 7%). The Harman approach overestimates the roof absorption, which is expected as the single-layer infinite street approach neglects roof shadowing.

Overall, our results show the SPARTACUS multi-layer approach to modelling radiative transfer in urban areas agrees well with the more complex and computationally demanding radiative transfer model DART when modelling real-world cities. The SPARTACUS-Urban model is the first multi-layer urban radiation model to achieve this, whilst being computationally cheap enough to be incorporated into weather and climate models (Hogan 2019a). This work surpasses previous evaluations that compare radiative transfer models to small scale observations or to more simplistic radiative transfer models (e.g., Harman et al. 2004; Krayenhoff and Voogt 2007; Aoyagi and Takahashi 2012; Krayenhoff et al. 2014). The single-exponential distribution that underpins SPARTACUS-Urban performs well but can be improved by using a two-exponential method. It is not yet certain if the extra complexity of implementing this two-exponential is justified, given the uncertainty in urban morphology datasets. Such datasets are required to compute required model inputs for SPARTACUS-Urban to describe the urban form (i.e., vertical descriptions of the urban canopy), which would be required if SPARTACUS-Urban is to be applied into a large-scale model.

Further investigation is needed to ascertain the amount of data required to describe building geometry worldwide, and how this impacts radiative fluxes. Further evaluation should be completed with SPARTACUS-Urban in the longwave, as this is a significant term in the urban surface energy balance (Oke 1988). As SPARTACUS-Urban can be integrated within existing urban surface energy budget models, the results of this shortwave evaluation provide a promising start to improving the treatment of the complex urban structure in NWP models.

### Electronic supplementary material

Below is the link to the electronic supplementary material.

Supplementary file1 (PDF 531 kb)

## Figures and Tables

**Fig. 13:**
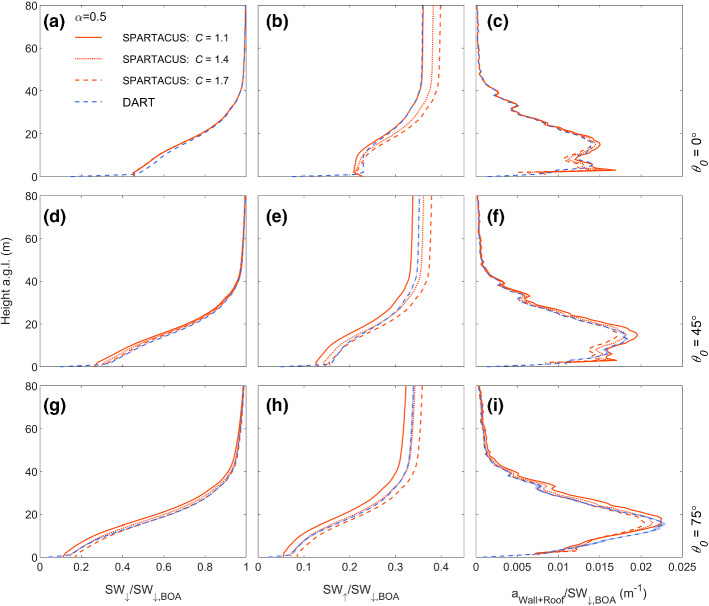
As Fig. 9, but SPARTACUS-Urban profiles indicating the variation from using a concavity parameter of 1.1 (solid), 1.4 (dotted), and 1.7 (dashed). SPARTACUS-Urban uses the two-exponential (Eq. 3) only

**Fig. 14:**
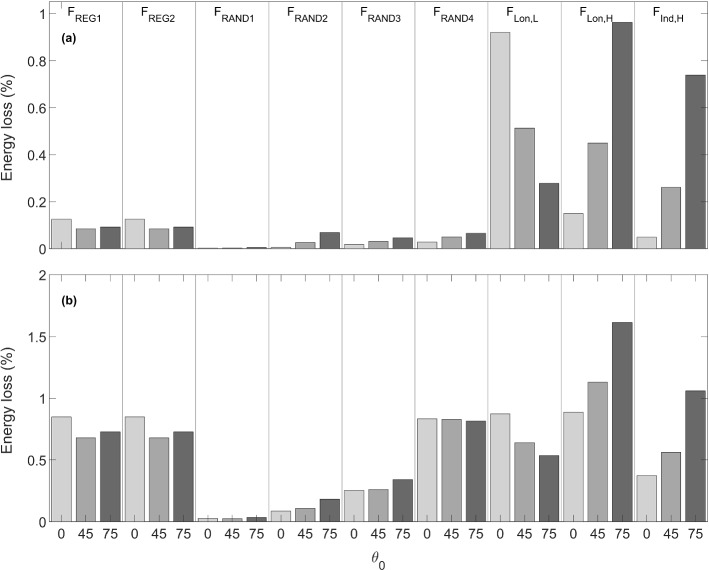
Energy loss (Eq. 16) in DART scenes (Table 1) for three solar zenith angles (*θ*_0_*:* 0°, 45°, 75°) for albedos: **a** 0.1, and **b** 0.5, for all F_REG_, F_RAND1-4_, and real-world (F_Lon,L_, F_Lon,H_, F_Ind,H_) scenes tested (Sect. 4.1)

**Fig. 15:**
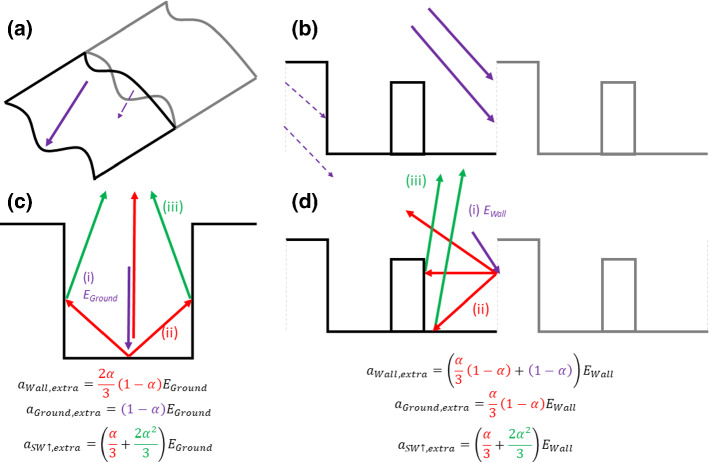
Energy lost in DART occurs at the edge of periodic model domains (grey) if there is **a** unmatched topography, and/or **b** buildings missing external walls. To distribute the energy through the **c** ground (*E*_Ground_), and **d** walls (*E*_Wall_), where the purple arrows (i) represent the extra energy into the ground and walls, and the red (ii) and green (iii) represent the first and second reflections of shortwave radiation, to determine the total extra shortwave radiation into the walls, ground, and upwelling (*a*_Wall*,*extra_, *a*_Ground*,*extra_, and SW_↑,extra_)

## Appendix 1: Building Concavity in Real-World Cities

As outlined in Sect. 2.2, a correction is made in SPARTACUS-Urban to account for the building concavity in real-world cities. Real-world buildings tend to not be convex, which means the relation between the building perimeter length given to SPARTACUS-Urban and the rate of radiation exchange is not correct. Therefore, we correct for this concavity by applying a constant scaling factor to *L* at each height level using a concavity parameter (*C,* Eq. 11). This allows us to approximately obtain the perimeter of the convex hull of each building. Here, three real-world domains are analyzed (in London and Indianapolis) at two levels of detail (LOD, low and high) (F_Lon,L_, F_Lon,H_, F_Ind,H_, Table 1), derived from building footprints (Table 1).

For the London domains, *C* ranges between 1.04 and 1.66 at each height interval, with smaller values for the low (F_Lon,L_) than high (F_Lon,H_) LOD domain (Fig. 12). Generally, *C* decreases with height in real cities (Fig. 12, Online Resource 7), because individual buildings in an area of the city may be short and wide, or tall and narrow, and so cast different sized shadows. Cross-sections of the high LOD London domain support this, with low height levels, where the building density is high, having a larger *C* (cf. higher heights where smaller, taller building occur) (Online Resource 7d). Given this, we calculate *C* at all heights, and use median *C* from all heights above $\overline{H }$, applying this as the scaling factor to *L*(*z*) (black, Fig. 12). Values below $\overline{H }$ are excluded given both the presence of courtyards, and that taller buildings will shadow shorter buildings (rather than vice versa).

We examine the impact on the shortwave fluxes, with *C* from 1.1 to 1.7 but constant with height (Sect. 4.3). Across the three solar zenith angles (Fig. 13), the *C* value has the greatest impact on the SW_↑_ profiles for the F_Lon,H_ domain, and less impact on the SW_↓_ and *a*_Wall+Roof_ profiles.

## Appendix 2: Redistribution of Lost Energy in the DART Model Output

We calculate energy imbalance (*Ε*, W m^−2^/W m^−2^) for each DART run, using

16 $$ \begin{array}{*{20}c} {{E} = 1 -\left( {a_{{{\text{Wall}}}} + a_{{{\text{Roof}}}} + a_{{{\text{Ground}}}} + {\text{SW}}_{{ \uparrow , {\text{top}}}} } \right)} \\ \end{array} $$

where the total wall, ground and roof absorption are given by *a*_Wall_, *a*_Ground_ and *a*_Roof_, respectively and SW_↑,top_ is the shortwave upwelling clear air flux at the top of the canopy. *Ε* = 0 for a perfectly conserving model.

The DART energy imbalance is always less than 2%, and usually less than 1% (Fig. 14b). Generally, energy conservation is highest for overhead sun conditions (*θ*_*0*_ = 0°) and decreases with geometry complexity to be lowest for the real-world domains (F_Lon_, F_Ind_). F_REG_ (Table 1) domains have a lower voxel resolution, so do not follow this relation, as DART energy conservation tends to improve as voxel resolution increases (e.g. 1 m → 0.5 m).

Processes identified that contribute to energy loss are (Fig. 15):

1. rays passing ‘underground’ of the domain when there are holes in the 3D building model,
2. rays hitting internal walls of buildings

Both can occur from the domain periodic boundary conditions, if buildings and topography are cut at the domain edge (Wang et al. 2020). The F_RAND_, F_Lon,H_ and F_Ind,H_ (Table 1) 3D building models contain some buildings that cross the domain edge. As the domain cuts though it creates ‘open’ buildings allowing rays to pass directly underneath the building footprint model or interact with the ‘internal’ walls of the buildings (Fig. 15b). With non-flat topography (e.g., F_Lon,H_ and F_Ind,H_) the periodic boundary conditions create gaps through which rays can pass (Fig. 15a). F_REG_ scenes have neither of these features, whereas F_RAND_ scenes have only building split, hence energy loss increases with increasing domain complexity. As the real-world scenes have the largest energy loss, the DART missing energy is assumed to be lost through the building walls and the ground, attributed to processes (1) and (2) above.

For high LOD scenes, we redistribute lost energy into the walls (Fig. 15b, d) first as a ratio of total *A*_*W*_ to total a_Wall_. The approximated (*A*_*W,*edges_) wall area missing at the edge of the domain is used to calculate the average wall absorption

17 $$ \begin{array}{*{20}c} {a_{{{\text{Wall}}, {\text{edges}}}} = A_{{W,\,{\text{edges}}}} \frac{{a_{{{\text{Wall}}}} }}{{A_{W} }}.} \\ \end{array} $$

We note *A*_*W,*edges_ is an overestimate, as building walls in the repeated units may overlap. We assume *a*_Wall,edges_ is the amount of energy absorbed by these extra walls. The amount of total energy available for exchange by the wall processes (*E*_Wall_) in Fig. 15d is equal to ${a}_{\mathrm{Wall},\mathrm{edges}}/\left(1-\alpha \right)$. This energy is split between SW_↑_, *a*_Wall_, and *a*_Ground_ (Fig. 15d). The remainder of the lost energy is attributed to the ground process (*E*_Ground_) (Fig. 15a), and is redistributed (Fig. 15c) into *a*_Ground_, *a*_Wall_, and SW_↑_, where the two-thirds of the radiation reflected from the ground is distributed into *a*_Wall_. Combining these processes (Fig. 15) leads to the total added energy.

This is distributed at all height levels using a scaling factor (e.g., ${a}_{\mathrm{Wall}}/{a}_{\mathrm{Wall},\mathrm{extra}}$). For F_Lon,L_, the buildings at the edge of the domain have complete walls with flat topography, so we distribute lost energy through both the wall and ground processes (Fig. 15) equally, assuming *E*_Wall_ = *E*_Ground_. Although we redistribute the energy through these processes, we note this may not match the true DART results if external walls and topography are corrected. The uncertainty in these numbers is less than the range if solar azimuth angle is varied, and so it not shown in the vertical flux profiles compared in Sect. 6. In Sect. 7, this uncertainty is shown by an error bar across: the fluxes if no correction for the energy imbalance is made, and the fluxes if double the correction is made.
