# Supplementary material for: Evaluation of the SPARTACUS-Urban Radiation Model for Vertically Resolved Shortwave Radiation in Urban Areas
Source: Boundary Layer Meteorol. 2022 Jun 29;184(2):301–31. doi: 10.1007/s10546-022-00706-9 (PMC9259530; doi:10.1007/s10546-022-00706-9)
Supplement: Supplementary file 1 — Supplementary file1 (PDF 531 kb) [file 10546_2022_706_MOESM1_ESM.pdf]

Supplementary Material for Evaluation of the SPARTACUS-Urban Radiation Model for Vertically Resolved Shortwave Radiation in Urban Areas

Megan A Stretton* • William Morrison • Robin J Hogan • Sue Grimmond

*m.stretton@pgr.reading.ac.uk, Department of Meteorology, University of Reading, Reading, UK

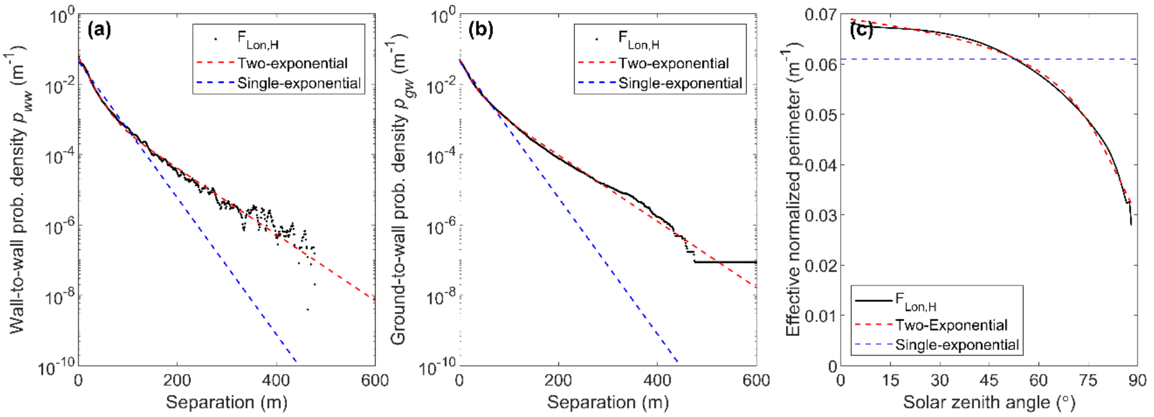

Online Resource 1 As Fig. 3,but for high level of detail central London ($F_{Lon,H}$)

Online Resource 2 As Table 2 but for α = 0.1

θ_0 (°)	Scene Albedo (z = 5.5 m)		a_{Ground}		a_{Wall}	
	DART	nBE (%)	DART	nBE (%)	nMAE (%)	nMBE (%)
(a) F_{REG1}						
0	0.0889	1.8	0.844	-0.011	20	-10
45	0.0869	0.59	0.779	0.44	11	0.78
75	0.0795	0.93	0.614	3.7	19	-7.1
(b) F_{REG2}						
0	0.0674	14	0.676	-0.044	30	-23
45	0.0620	13	0.411	21	35	-26
75	0.0590	2.6	0.170	30	31	-11

Online Resource 3 As Table 3, but for the remainder of F_{RAND} scenes tested, with $\overline{H}$ = 7, 15, 25 m, λ_p = 0.05, 0.15 0.35, 0.5

F_{RAND}	$\lambda_{p,0}$	$\overline{H}$ (m)	H_{max} (m)	Scene Albedo (z = H_{max})		a_{Ground}		a_{Wall}		a_{Roof}	
				DART	nBE (%)	DART	nBE (%)	nMAE (%)	nMBE (%)	nMAE (%)	nMBE (%)
5	0.05	15	34	0.466	-0.75	0.426	-0.61	3.0	2.8	2.2	0.71
6	0.5	15	59	0.362	-4.2	0.0673	-5.8	5.4	5.3	4.6	0.20
7	0.15	7	22	0.456	-1.6	0.360	-1.1	2.7	2.6	0.97	0.46
8	0.15	15	61	0.414	-2.0	0.301	-1.5	3.5	3.4	2.0	0.90
9	0.15	25	85	0.373	-2.4	0.237	-0.57	3.0	2.9	3.4	-0.77
10	0.35	7	27	0.420	-3.0	0.210	-2.7	3.8	3.8	2.1	0.43
11	0.35	15	45	0.367	-3.8	0.136	-3.3	4.4	4.4	2.2	0.40
12	0.35	25	83	0.329	-4.1	0.0817	-2.6	4.4	4.4	3.3	-0.040

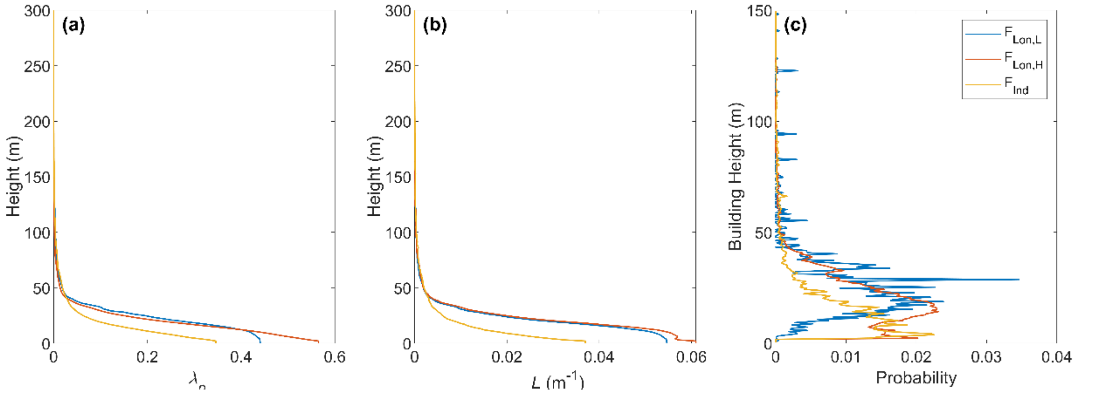

Online Resource 4 Vertical profiles of (a) building fraction (λ_p), (b) normalised building edge length (L), and (c) distribution of building heights, for central London domains ($F_{Lon,L}$, $F_{Lon,H}$) and Indianapolis (F_{Ind}) (Table 1)

Online Resource 5 As Table 4, but for α = 0.1

θ_0 (°)	Scene Albedo (z = H_{max})			a_{Ground}			a_{Wall}				a_{Roof}			
	DART	Single	Two	DART	Single	Two	Single	Two	Single	Two	Single	Two	Single	Two
		nBE (%)			nBE (%)		nMAE (%)		nMBE (%)		nMAE (%)		nMBE (%)	
(a) $F_{Lon,L}$														
0	0.0697	-1.6	-4.7	0.502	0.37	0.39	18	12	-16	-10	7.6	7.6	0.94	0.94
45	0.0671	-9.3	-9.9	0.265	7.4	5.7	3.5	3.5	-0.76	0.95	10	10	-4.2	-4.3
75	0.0617	-13	-9.9	0.0861	-24	4.9	10	5.0	10	3.6	17	14	-9.4	-6.9
θ_0 (°)	Scene Albedo (z = H_{max})			a_{Ground}			$a_{Wall+Roof}$							
	DART	Single	Two	DART	Single	Two	Single				Two			
		nBE (%)			nBE (%)		nMAE (%)		nMBE (%)		nMAE (%)		nMBE (%)	
(b) $F_{Lon,H}$														
0	0.0643	5.9	3.6	0.424	-7.5	-7.5	10		5.7		10		5.9	
45	0.0618	-2.3	-3.0	0.266	-21	-22	9.9		8.2		10		8.7	
75	0.0576	-8.5	-5.7	0.0902	-53	-36	15		6.0		14		4.0	
(c) $F_{Ind,H}$														
0	0.0800	1.6	0.38	0.610	-3.4	-3.4	15		6.9		15		7.1	
45	0.0779	-3.3	-4.1	0.525	-9.0	-9.8	15		12		15		14	
75	0.0699	-7.9	-7.3	0.351	-22	-21	17		14		17		13	

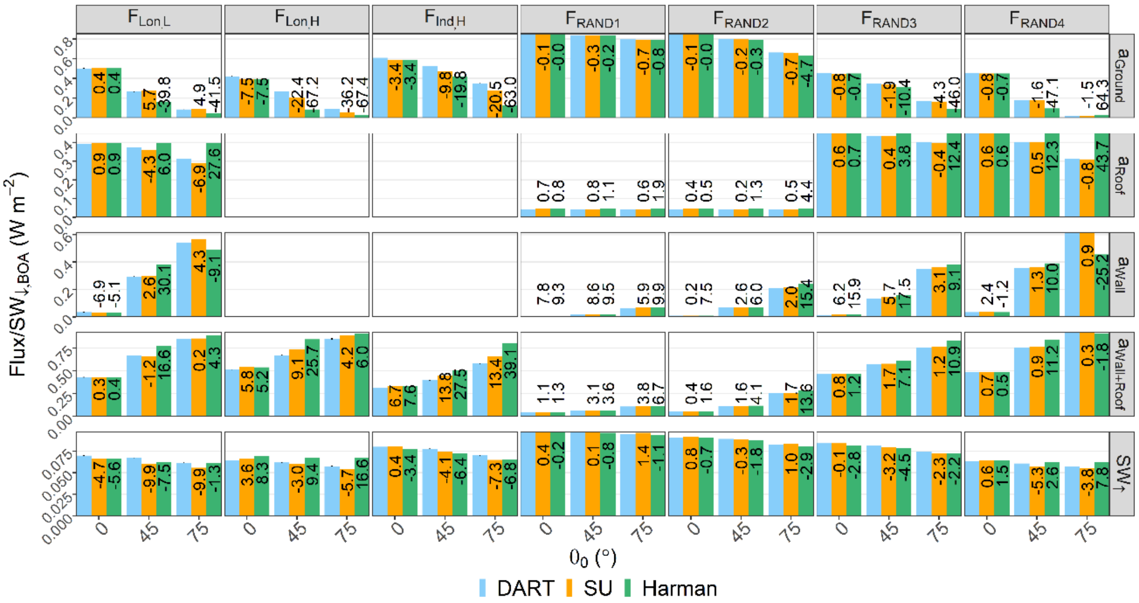

Online Resource 6 As Fig. 11 but for α = 0.1

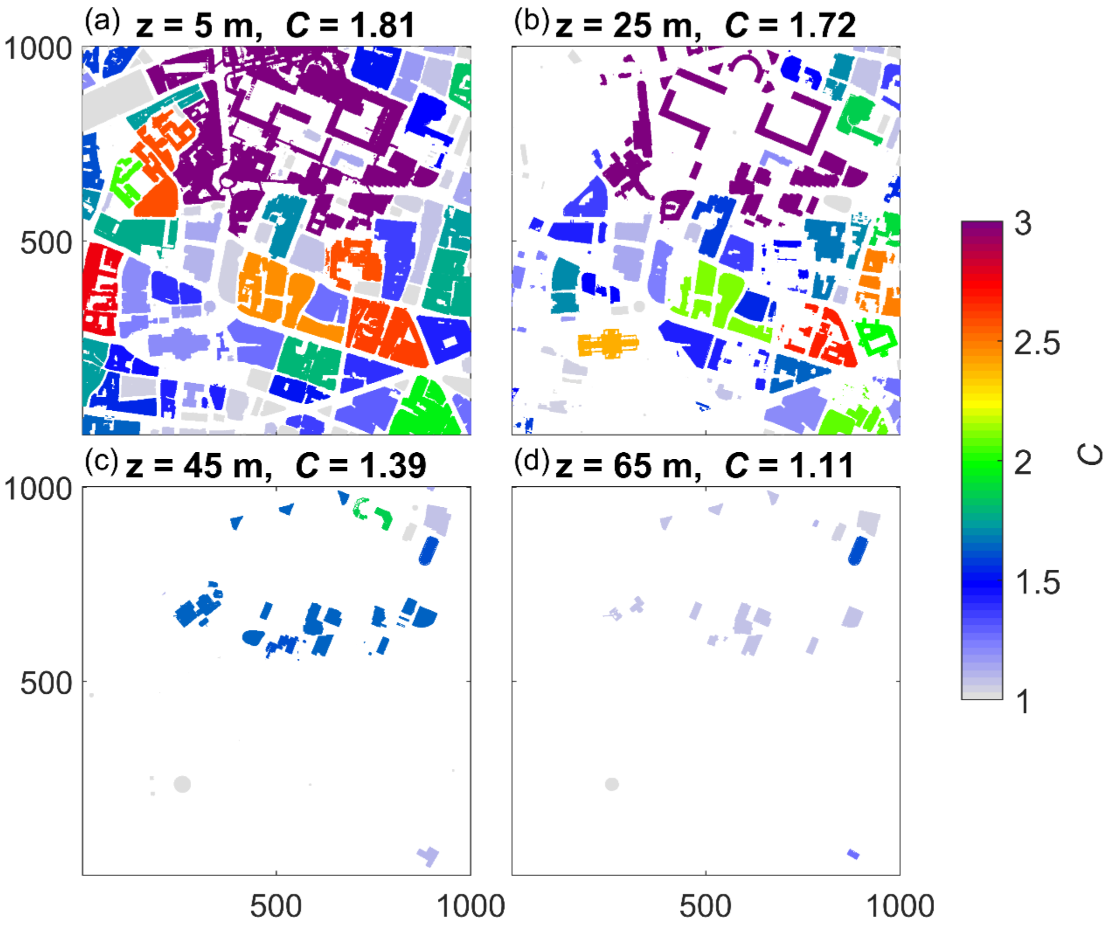

Online Resource 7 Variation of the concavity parameter (*C*, Eq. 11) for slices of individual buildings in a subset (1000 × 1000 m) of the high level of detail London scene, at four height levels: (a) 5 m, (b) 25 m, (c) 45 m, (d) 65 m
